# The interactions between sleep difficulties and clinical features in children with rare genetic syndromes: a structural equation modelling approach

**DOI:** 10.1186/s12887-026-07099-5

**Published:** 2026-06-05

**Authors:** Rory O’Sullivan, Paul Thompson, Stacey Bissell, Andrew P. Bagshaw, Kelly Wade, Effie Pearson, Caitlin Murray, Kayla Smith, Elizabeth Berry, Hayley Crawford, Joanna Moss, Jane Waite, Caroline Richards

**Affiliations:** 1 School of Psychology, University of Birmingham, 52 Pritchatts Road, Birmingham, B15 2TT UK; 2 Cerebra Network for Neurodevelopmental Disorders, Surrey, Warwick, Birmingham, UK; 3 Health Services Management Centre, University of Birmingham, Birmingham, UK; 4 Centre for Human Brain Health, University of Birmingham, Birmingham, UK; 5 College of Health and Life Sciences, Aston University, Birmingham, UK; 6 Autistica, London, UK; 7 Department of Education, University of Oxford, Oxford, UK; 8 Warwick Medical School, Mental Health and Wellbeing Unit, University of Warwick, Coventry, UK; 9 School of Psychology, University of Surrey, Guildford, UK

**Keywords:** Sleep, Intellectual disability, Genetic syndrome, Children, Behaviours that challenge, Mental health, Autism, ADHD, Epilepsy, Socioeconomic status

## Abstract

**Background:**

Sleep difficulties are common in children with rare genetic syndromes and are associated with clinical features such as behaviours that challenge and mental health difficulties. This study aimed to further clarify the role of sleep difficulties in children’s clinical outcomes by (1) describing profiles of sleep difficulties in syndromes that had received little or no detailed sleep investigation, such as KBG and Kleefstra syndrome; (2) statistically controlling the interactions between clinical features when examining the associations with sleep difficulties; and (3) exploring the associations between sleep difficulties and demographic characteristics. Overall, this study aimed to describe the profiles of sleep difficulties experienced within genetic syndromes and examine the associations between sleep difficulties, clinical features, and demographic characteristics.

**Methods:**

Caregivers of children with rare genetic syndromes (*N* = 251, aged 1–18 years) completed questionnaires that assessed sleep difficulties and various clinical features. Profiles of sleep difficulties were described by syndrome and compared via odds ratios to typically-developing children. Structural equation modelling was used to examine the associations between sleep difficulties, clinical features, and demographic characteristics, whilst controlling for interactions between clinical features.

**Results:**

Across most syndromes, night wakings, bedtime resistance, sleep-disordered breathing, parasomnias, and sleep duration difficulties were elevated compared to typically-developing children, although several syndrome-specific profiles of sleep difficulties were also observed. Across syndromes, sleep difficulties were significantly, positively associated with anxiety and depressed mood, autism-related characteristics, overactivity and impulsivity, epilepsy severity, and the severity of physical health conditions. Of the demographic characteristics, sleep difficulties were associated with socioeconomic status.

**Conclusion:**

These findings have several implications. Firstly, the profiles of sleep difficulties described in syndromes with little prior sleep investigation (e.g. Coffin-Siris, KBG, and Kleefstra syndrome) highlights the need to investigate sleep in more detail in these groups and further informs the phenotypes of the syndromes. Secondly, sleep difficulties were associated with numerous clinical features, even after controlling for other predictors, highlighting the importance of sleep as a clinically modifiable variable that could improve clinical outcomes in rare syndromes. Thirdly, the association between sleep difficulties and socioeconomic status highlights the need to examine environmental determinants of poor sleep in children with rare genetic syndromes.

**Supplementary Information:**

The online version contains supplementary material available at 10.1186/s12887-026-07099-5.

## Introduction

Poor sleep is common in rare genetic syndromes associated with intellectual disability (ID) [1]. Studies of these syndromes, many of which focus on children, have consistently demonstrated that poor sleep is associated with clinical features such as behaviours that challenge (BtC) [2–7], depression and anxiety [2, 8–10], physical health conditions [6, 7, 11], and autism [7, 12–14] and ADHD characteristics [2, 11, 15–17]. These clinical features are themselves common in many syndrome groups [18–21], and, alongside poor sleep, can be associated with substantial burden for children and their families [22–25]. Further examination of the associations between sleep and broader clinical features in children with rare genetic syndromes is essential to understand the role of sleep within children’s broader clinical outcomes and delineate putative mechanisms to improve child and family outcomes.

Whilst poor sleep has been identified in many rare genetic syndromes associated with ID, the precise profiles of sleep difficulties remain poorly described in many groups. The lack of descriptive data can be attributed to several factors, including (i) reliance on case studies, such as in KBG syndrome [26, 27], (ii) use of non-standardised sleep measures, such as in Kleefstra syndrome [28], and (iii) absence of studies investigating specific sleep difficulties, such as in Coffin-Siris syndrome. Even for syndromes that have been assessed with standardised sleep measures and relatively large samples, descriptions of sleep difficulties experienced by children are sparse and could be ratified with further data. Examples of these syndromes include Pallister-Killian [29, 30], Wiedemann-Steiner [31], and SATB2-associated syndromes [32]. Precise description of the topography and severity of sleep problems experienced by children and, separately, adults with these syndromes is necessary to advance phenotypic description and target sleep interventions [33].

As mentioned, poor sleep has been empirically and theoretically associated with numerous clinical features in rare genetic syndromes associated with ID and there is emerging evidence that these clinical features are also related to each other. For example, positive cross-sectional associations have been noted between (i) painful health conditions and BtC [34–36], (ii) BtC and anxiety and depressed mood [37–39], (iii) autism-related characteristics and ADHD-related characteristics such as overactivity/impulsivity [40–42], and (iv) epilepsy severity and non-verbal status [43–45]. Additionally, biological factors associated with rare genetic syndromes, such as craniofacial differences [46, 47], muscular hypotonia [48] and atypical melatonin release patterns [49, 50], can impact sleep quality, quantity, and timing. Altogether, the evidence suggests a complex network of interactions between sleep and clinical features in rare genetic syndromes, likely comprised of direct and indirect pathways (see [51, 52]). However, these pathways between clinical features have rarely been accounted for in previous sleep studies. Accounting for interactions between clinical features is essential to estimate the relevance of sleep difficulties to children’s broader outcomes and hence to understand the potential role of sleep as a modifiable variable to improve children’s outcomes.

In addition to clinical features, research with typically-developing (TD) children has demonstrated that sleep difficulties are associated with demographic characteristics such as age, sex, socioeconomic status (SES) and ethnicity [53–57]. Although associations between demographic characteristics and sleep difficulties have been examined in rare genetic syndromes, these predominantly relate to age and sex [8, 11, 17, 58, 59]. Given that ethnicity [60] and SES [61] have been strongly associated with sleep quantity and quality in TD populations, these characteristics should be examined to obtain a comprehensive account of the factors that impact sleep in rare genetic syndromes.

In summary, research is needed to: (i) delineate profiles of sleep difficulties in children with rare genetic syndromes associated with ID, especially those with little-to-no existing description; (ii) control for the interactions between clinical features when examining the associations between clinical features and sleep difficulties; and (iii) explore whether demographic characteristics are associated with sleep difficulties in rare genetic syndromes. Therefore, the aims of this study were to^1^:

1. Explore differences in sleep difficulties between children with rare genetic syndromes and TD children, including syndromes with little robust description of sleep difficulties.
2. Examine the interactions between sleep difficulties and clinical features in children with rare genetic syndromes, whilst controlling for interactions between clinical features.
3. Explore whether associations between demographic factors and sleep difficulties observed in TD populations are replicated in children with rare genetic syndromes.

## Methods

This study was pre-registered prior to data collection on the Open Science Framework (https://osf.io/5tzv7).

### Participants

This study focuses on a subset of children, diagnosed with rare genetic syndromes associated with ID, from a larger survey study titled ‘Behavioural and Emotional Outcomes in Neurodevelopmental Disorders study’ (BEOND; https://osf.io/n89x7*).* All children were aged between 1 and 18 years and their caregivers had sufficient English language abilities to understand the informant-report questionnaires. Caregivers were recruited using databases held at institutions across the Cerebra Network for Neurodevelopmental Disorders as well as social media posts, syndrome support groups, and specialist schools.

In total, caregivers of 353 children with rare genetic syndromes were recruited. Listwise deletion resulted in the exclusion of 102 children with incomplete data (i.e. at least one of the questionnaires described below was left incomplete). An additional 13 children were removed as the sleep and clinical outcome questionnaires had been completed ≥ 30 days apart (see study procedure below), leaving a final sample of 238. Although recruited across 19 countries, most caregivers in the final sample were recruited from the United Kingdom (*N* = 121) and United States of America (*N* = 65). The number of caregivers recruited from each country are reported comprehensively in Additional file 1.

### Measures

Questionnaires relevant to the pre-registered analyses are described below^2^.

#### Background information

##### Background questionnaire

The background questionnaire was a study-specific measure of demographic characteristics, including age, sex, ethnicity, SES, verbal ability, and level of ID. Most variables were categorical and the corresponding definitions/response options are presented in Additional file 3.

#### Sleep

##### Children’s Sleep Habits Questionnaire (CSHQ) [62]

The CSHQ was used to measure the frequency of sleep-related behaviours within the last week. The CSHQ is frequently used in studies of rare genetic syndromes, being the most common questionnaire to assess insomnia-related difficulties (sleep onset delay/sleep duration/night awakenings) in a systematic review and meta-analysis of 273 studies of 55,310 individuals with rare genetic syndromes [1]. The CSHQ was administered to the entire sample, regardless of age, as this is common practice in studies of rare genetic syndromes (e.g. [8, 63, 64]). Thirty-one items are scored on a 3-point scale ranging from “usually, 5–7 times in the last week” (3) to “rarely, 0–1 times in the last week” (1), and two items were scored on a 3-point scale ranging from “Falls asleep” (3) to “Not sleepy” (1). Scores from the 33 items are combined into a total score and eight subscale scores (outlined below). Higher scores indicate greater severity of sleep difficulties. In the current sample, Cronbach’s alpha indicated moderate-to-good internal consistency for the bedtime resistance (α = 0.76), sleep duration (α = 0.79), sleep anxiety (α = 0.62), night awakenings (α = 0.64), parasomnias (α = 0.57), sleep-disordered breathing (α = 0.73), and daytime sleepiness (α = 0.67) subscales. Subscale scores > 2 standard deviations above published means [62] were considered indicative of clinically-significant sleep problems [65–69].

#### Behaviour

##### The Activity Questionnaire (TAQ) [70]

The TAQ was used to assess the frequency of behaviours related to overactivity, impulsivity, and impulsive speech within the last month. Eighteen items are rated on a five-point Likert-type scale ranging from ‘Never/almost never’ (0) to ‘Always/almost all the time’ (4). A total score can be calculated alongside subscale scores for ‘overactivity’, ‘impulsivity’, and ‘impulsive speech’, with higher scores reflecting a greater frequency of the associated behaviours. Cronbach’s alpha revealed that the overactivity and impulsivity subscales, used in this study, had excellent internal consistency in the current sample (overactivity: α = 0.91, impulsivity: α = 0.91).

##### The Challenging Behaviour Questionnaire (CBQ) [71]

The CBQ assesses whether self-injurious, aggressive, destructive, and stereotyped behaviours have occurred (i) in the last month and (ii) at any point in an individual’s life. For behaviours that had occurred in the previous month, caregivers rate the duration, frequency, and necessity of restraint/preventative measures. Composite severity scores are calculated from these ratings, ranging from 0 to 14, with higher scores indicating greater severity. Good inter-rater reliability has been demonstrated for the self-injury (κ = 0.92), physical aggression (κ = 0.85), destruction of property (κ = 0.75), and stereotypic behaviour subscales (κ = 0.60) in a previous rare genetic syndrome sample [71]. In the current sample, Cronbach’s alpha demonstrated moderate-to-good internal consistency for the self-injury (α = 0.55), physical aggression (α = 0.71), and destruction of property (α = 0.65) subscales^3^.

#### Autism-related characteristics

##### Social Communication Questionnaire - Current (SCQ) [72]

The SCQ is a screening measure for autism based on the Autism Diagnostic Interview, administered for children over 4 years old. Respondents provide “yes” (1) or “no” (0) answers to 40 items, indicating the absence or presence of autism characteristics over the previous 3 months. A total score was produced, ranging from 0 to 39 for verbal individuals and 0–33 for non-verbal individuals. The measure has good diagnostic validity, distinguishing between children with ID and pervasive developmental disorders with sensitivity and specificity values of 0.92 and 0.62, respectively [73]. The measure also has good convergent validity with the Autism Diagnostic Interview-Revised (*χ*^2=^19.29; [74]) and, in the current sample, Cronbach’s alpha indicated good internal consistency of the total scores (α = 0.82).

##### Modified Checklist for Autism in Toddlers - Revised (M-CHAT-R) [75]

The M-CHAT-R is a screening measure for autism based on the Checklist for Autism in Toddlers. Although originally designed for children between 16 and 30 months of age, the M-CHAT-R was administered to all children < 4 years of age in the current study. This decision was informed by the relatively good sensitivity and specificity of the M-CHAT-R for children within (16–30 months) and outside (31–45 months) of the validated age range [76]. The 20-item measure assesses the presence of symptoms associated with autism throughout a child’s life, with each item scored as “yes” (0) or “no” (1). The number of item responses that indicate autism risk are counted, with 0–2 items suggesting low risk, 3–7 items suggesting medium risk, and 8–20 items suggesting high risk. The measure has good diagnostic validity in children at risk of developmental disorders, with sensitivity and specificity values of 0.73 and 0.66 [77]. The measure also had good internal consistency, according to Cronbach’s alpha, in the current sample (α = 0.87).

Since the SCQ and M-CHAT-R measure the same construct (autism-related characteristics) but were completed by different age groups, the total scores from each were converted to *z*-scores and combined into one autism variable.

#### Mental health

##### Clinical Anxiety Screen for people with Severe to Profound Intellectual Disability (ClASP-ID) [78]

The ClASP-ID is a 33-item questionnaire designed to assess anxiety in individuals with intellectual disabilities. The ClASP utilises a 7-point scale, ranging from 1 (“almost never”) to 7 (“all of the time/more than once a day”), to assess the frequency of behaviours associated with anxiety, pain, low energy/withdrawal, and consolability over the previous month. Subscale scores can be calculated for each of these behavioural categories, although only the anxiety subscale was used for the current study. Scores for the anxiety subscale range from 14 to 98, with higher scores indicating greater anxiety. The anxiety subscale has good convergent and divergent validity, robust internal consistency (α = 0.92), good test-retest reliability (ICC = 0.88), and moderate inter-rater reliability (ICC = 0.64) for individuals with intellectual disabilities [78]. Cronbach’s alpha also indicated that the subscale had robust internal consistency in the current sample (α = 0.88).

Mood Interest and Pleasure Questionnaire (

##### Mood Interest and Pleasure Questionnaire (MIPQ) [79]

The MIPQ contains 12 items designed to measure the frequency of behaviours associated with low mood and interest and pleasure in the previous two weeks, using a 5-point scale. Ratings are compiled into total scores (ranging from 0 to 48) with lower scores indicating lower mood, interest, and pleasure. The total scores have excellent test-retest (*r =* 0.97) and inter-rater reliability (*r* = 0.85) amongst samples of rare genetic syndromes [80]. Good internal consistency was also demonstrated for the total scores, via Cronbach’s alpha, in the current sample (α = 0.84).

#### Physical health

##### Health Questionnaire (HQ) [81]

The HQ examines the presence and severity of 15 physical health difficulties, with two sections specific to lifetime occurrence and occurrence in the preceding month. The presence and severity of health difficulties in the preceding month are rated on a 4-point scale, ranging from 0 (“Never”) to 3 (“Severe”). Total health problem scores are calculated from these ratings, ranging from 0 to 48, with higher scores indicating more severe profiles of health conditions. Good inter-rater reliability has been observed for total health problem scores (κ = 0.65) in a previous rare genetic syndrome sample [81].

##### Hague Seizure Severity Scale (HSSS) [82]

The HSSS was only administered when caregivers indicated, via the HQ, that children had experienced seizures within the previous month. Respondents rate the frequency, duration, or severity of 13 ictal and postictal problems in the previous 3 months. A 4-point scale is used for 11 items, and a 5-point scale for the remaining two (adding a “does not apply” response option). Item responses are summated into a total score ranging from 13 to 54, with higher scores indicating greater severity. The scale was partially developed from items in the Liverpool Seizure Severity Scale [83] and has good test-retest reliability in children with epilepsy (*r =* 0.93; [82]). Cronbach’s alpha indicated that, in the current sample, the total scores had robust internal consistency (α = 0.87).

### Procedure

Data collection took place as part of the BEOND study, conducted across the Cerebra Network research teams at the University of Birmingham, Aston University, University of Surrey and the University of Warwick. During 15 months of data collection (November 2022 to January 2024), caregivers completed the questionnaires via an online survey, hosted by REDCap software [84] or via posted paper surveys. In instances where the online survey was started but not completed, and where posted surveys were not returned, caregivers were contacted and prompted to complete the survey. Caregivers could provide documentation confirming children’s genetic syndrome diagnoses (e.g. letters from relevant healthcare professionals) but this was not mandatory for participation. These documents were received for 92/238 children in the final sample and were in agreement with the diagnoses reported by caregivers [see Additional file 4].

### Analysis plan

Unless otherwise specified, all analyses were conducted using R version 4.2.1. The assumptions of parametric tests were assessed prior to analyses, and, when violated, non-parametric equivalents were used. All analyses were conducted following (i) listwise deletion (structural equation modelling) and pairwise deletion (remaining analyses), and (ii) multiple imputation. To reveal the degree to which the missing data impacted the results, the output of every statistical test was compared following listwise/pairwise deletion and multiple imputation. The assessment of missing data and the multiple imputation procedure are described in Additional file 5. The results following listwise/pairwise deletion are reported in the ‘Results’ section, those following multiple imputation are outlined in Additional file 6.

To address the first study aim, the proportion of children that scored above the CSHQ subscale cut-offs was identified in each syndrome and compared, via odds ratio tests, to published results from TD children aged 2–16 years without clinical sleep problems [67]^4^^, ^5. These comparisons were only conducted for syndromes with sufficient samples (*N* ≥ 10) after pairwise deletion and/or multiple imputation.

**Fig. 1:**
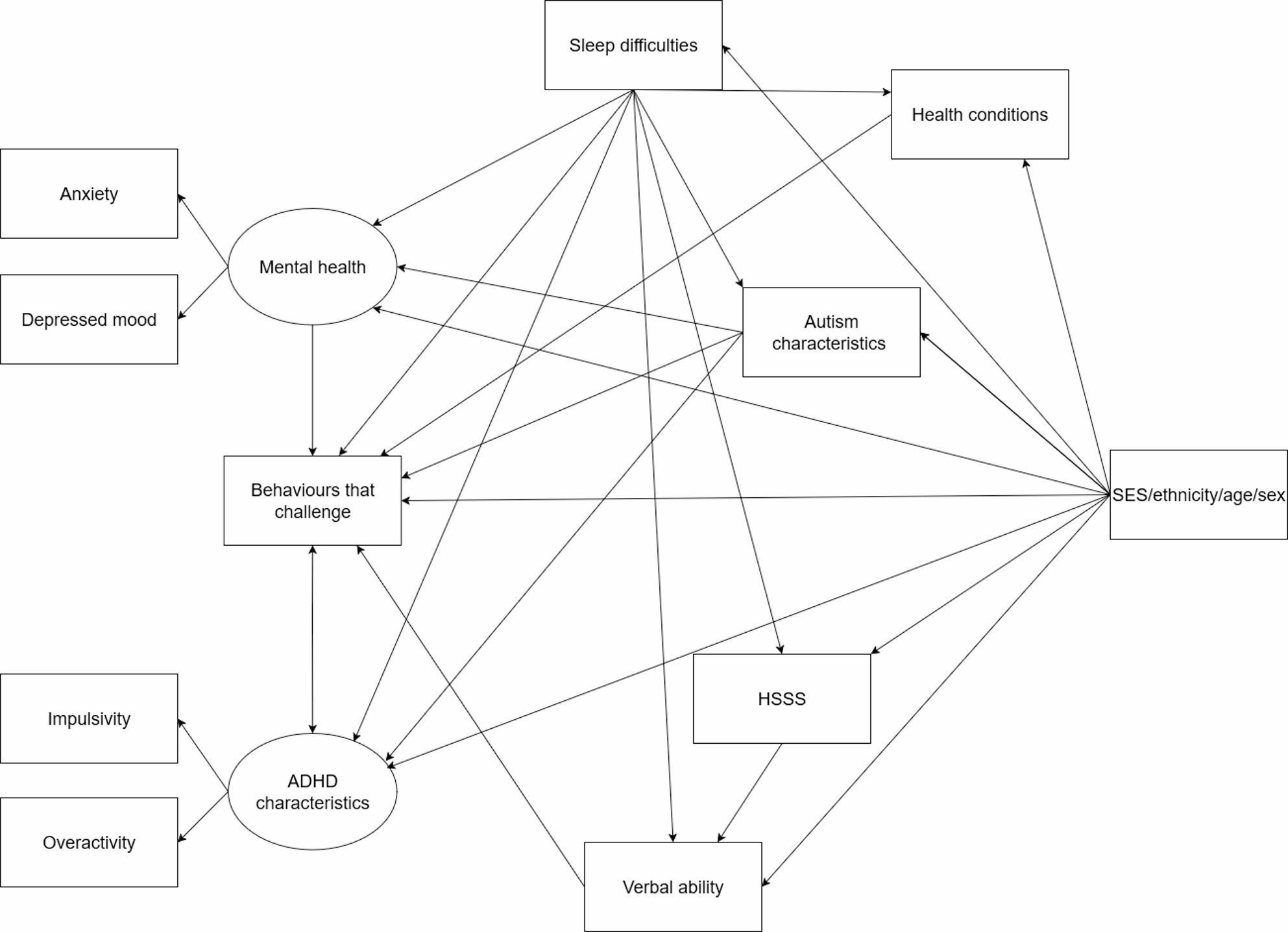
Graphical summary of the structural equation model. Note. ADHD: attention-deficit hyperactivity disorder. HSSS: Hague Seizure Severity Scale scores. SES: socioeconomic status. Rectangular variables represent observed variables, and oval variables represent latent variables. Arrows represent structural regression pathways, with the exception of arrows that represent factor loadings from observed variables to latent variables (e.g. impulsivity and hyperactivity loading onto ADHD characteristics). Although the arrows appear to visually indicate directionality, these represent regression analyses based on cross-sectional data and thus do not allow inferences of directionality. The demographic variables are compiled into one observed variable for the purposes of simplifying the diagram. Each demographic variable was separately regressed onto the sleep difficulties and clinical features, following the illustrated paths

To address the second study aim, structural equation modelling (SEM, see Fig. 1) was performed using the R *lavaan* package [85]. The SEM simultaneously examined associations between sleep, broad clinical features, and demographic characteristics, incorporating direct and indirect pathways informed by published findings and theory. This model was fitted for children across all genetic syndrome groups. Latent variables included: ‘mental health’ (CIASP-ID total and MIPQ total) and ‘attention-deficit hyperactivity disorder (ADHD) characteristics’ (TAQ impulsivity and overactivity subscales). The ‘ability’ and ‘externalising’ latent variables specified in the pre-registration were not included in the final analysis^6^. The ‘ability’ latent variable was substituted with verbal ability (directly measured in background questionnaire) as this has been more consistently associated with sleep difficulties in rare genetic syndromes [86–88] than adaptive functioning/intellectual ability [89, 90]. The ‘externalising’ latent variable was replaced by a BtC composite variable, calculated by summing scores for the self-injury, physical aggression, and destruction of property subscales. This composite was analysed as a binary variable, reflecting the absence (composite scores = 0) and presence (composite scores ≥ 1) of BtC. The HSSS scores were also zero-inflated and thus analysed as a binary variable, reflecting the absence (HSSS scores = 0) and presence (HSSS scores ≥ 1) of epilepsy. Additional binary variables included: verbal ability (0 = non-verbal, 1 = verbal), sex (0 = male, 1 = female), and ethnicity (0 = white (*n* = 201), 1 = mixed/black/Asian/other(*n* = 37)). Additional adjustments were also made to the SEM specified in the pre-registration. Firstly, demographic characteristics were added as control variables to the regressions between sleep and clinical features. Secondly, HSSS scores were regressed onto verbal ability, and covariance was estimated between ‘ADHD characteristics’ and BtC. These latter additions were informed by modification indices which show how the chi-square model fit statistic may change following the addition/removal of paths between model variables [91]. Notably, modifications were only made to the model if they were theoretically meaningful, such as the associations between HSSS scores and verbal ability [43–45] and overactivity/impulsivity and BtC [80, 92–94]. Collectively, these changes improved model fit and enhanced the inferences that could be drawn from the model.

The SEM was estimated using diagonally weighted least squares and the construct validity of the latent variables was assessed via factor loadings and squared multiple correlations (SMC; [95]). Model fit was assessed via the chi-square test (χ^2^), comparative fit index (CFI), Tucker-Lewis Index (TLI), root mean square error of approximation (RMSEA), standardised root mean square residual (SRMSR), and weighted root mean square residual (WRMSR). Compared to standard criteria for acceptable fit, less conservative criteria were applied given reductions in fit associated with SEM complexity [96]: χ^2^ *p* > 0.05, CFI ≥ 0.90, TLI ≥ 0.90, RMSEA ≤ 0.08, and SRMSR < 0.08 [97, 98].

The current sample (*N* = 238) was potentially insufficient for an adequately powered SEM, however, the sample following multiple imputation (*N =* 340) provides additional statistical power and any differences in estimates between imputed and unimputed samples offer some guide on the robustness of reported estimates. Sample sizes similar to that following multiple imputation have been employed throughout previous studies with SEMs that share similar properties to the current model [99–101]. Previous simulations also indicate that, for SEMs with these properties, 300 participants are sufficient to produce admissible models with minimal bias [102].

In addition to the pre-registered analyses, associations between sleep difficulties and different BtC topographies were also assessed. These exploratory analyses were included based on evidence that different BtC may be inconsistently associated with sleep difficulties [7, 81]. First, binary variables were coded from the zero-inflated aggression, self-injury, and destruction of property CBQ subscale scores, reflecting the absence (subscale score = 0) and presence (subscale score ≥ 1) of BtC. Logistic regressions were then conducted for each BtC topography, including the same predictors as the SEM. The results of these regressions are comprehensively reported in Additional file 7.

The third study aim was also addressed by the SEM presented in Fig. 1, as this regressed demo3graphic characteristics (age, sex, ethnicity, and socioeconomic status) onto sleep difficulties. To minimise type I error incurred by multiple comparisons, the false discovery rate was controlled at 10% via the Benjamini-Hochberg technique. This technique limits Type II errors compared to other corrections [103], thus minimising the likelihood of false negatives. No *p*-value correction was applied to the SEM analyses as, for models estimated from samples *N* ~ 200, even the least conservative correction methods severely reduce the power of individual parameters [104].

## Results

The demographic characteristics and level of ID in the final sample are described in Tables 1 and 2, respectively. The CSHQ descriptive statistics outline the profiles of sleep difficulties per syndrome (see Table 3).

**Table 1:** Demographic characteristics of the final sample, separated by genetic syndrome

Syndrome *(N)*	Age in years M*(SD)*	Male/ female (*N*)	Ethnicity					Socioeconomic status			
			Wh (*N*)	Mi (*N*)	Asi (*N*)	Bl (*N*)	Oth (*N*)	< NA (*N*)	~NA (*N*)	> NA (*N*)	ND (*N*)
AS *(13)*	13.29 *(3.92)*	4/9	9	4	0	0	0	4	1	8	0
CdCS *(5)*	6.07 *(3.75)*	1/4	4	0	1	0	0	0	2	3	0
CdLS *(16)*	8.88 *(3.06)*	10/6	12	1	2	1	0	3	5	6	2
CSS *(7)*	6.24 *(3.12)*	2/5	3	3	0	0	1	2	1	4	0
DS *(2)*	7.00 *(7.66)*	2/0	2	0	0	0	0	0	0	2	0
FXS *(22)*	11.37 *(4.15)*	20/2	18	0	2	1	1	3	0	18	1
JdVS *(7)*	8.94 *(3.17)*	4/3	7	0	0	0	0	1	1	4	1
KBGS *(30)*	7.69 *(4.27)*	20/10	27	2	1	0	0	6	10	13	1
KS *(19)*	9.10 *(5.21)*	9/10	18	1	0	0	0	4	2	11	2
PKS *(5)*	6.70 *(5.08)*	2/3	5	0	0	0	0	2	1	1	1
PWS *(9)*	6.23 *(4.57)*	6/3	7	1	0	0	1	4	0	4	1
RTS *(23)*	9.04 *(5.54)*	9/14	21	2	0	0	0	3	4	16	0
SAS *(29)*	9.42 *(3.99)*	17/12	23	4	2	0	0	6	4	16	3
SMS *(14)*	11.21 *(5.02)*	10/4	14	0	0	0	0	2	3	8	1
SYNGAP1 *(2)*	11.83 *(4.36)*	1/1	1	0	0	1	0	0	1	1	0
TSC *(9)*	9.05 *(5.03)*	5/4	7	2	0	0	0	2	1	6	0
WHS *(5)*	7.05 *(2.65)*	2/3	5	0	0	0	0	0	0	4	1
WSS *(6)*	11.28 *(4.69)*	2/4	6	0	0	0	0	1	0	4	1
Other *(15)*^*a*^	7.20 *(4.36)*	8/7	14	1	0	0	0	3	3	9	0
Total *(238)*	8.99 *(4.70)*	134/ 104	203	21	8	3	3	46	39	138	15

*M *mean, *Wh *White, *Mi *Mixed ethnicity or multiple ethnic groups, *Asi *Asian or Asian British, *Bl *Black, African, Caribbean, or Black British, *Oth *Other, *NA *national average household income, *ND *Not disclosed, *AS *Angelman syndrome, *CdCS *Cri-du-Chat syndrome, *CdLS *Cornelia de Lange syndrome, *CSS *Coffin-Siris syndrome, *DS *Down syndrome, *FXS *Fragile X syndrome, *JdVS *Jansen-de Vries syndrome, *KBGS *KBG syndrome, *KS *Kleefstra syndrome, *PKS *Pallister-Killian syndrome, *PWS *Prader-Willi syndrome, *RTS *Rubinstein-Taybi syndrome, *SAS *SATB2-associated syndrome, *SMS *Smith-Magenis syndrome, *TSC *tuberous sclerosis complex, *WHS *Wolf-Hirschhorn syndrome, *WSS *Wiedemann-Steiner syndrome

^a^ The ’Other’ group includes 12 distinct syndromes. Details of these syndromes and syndrome-specific demographics are in Additional file 2

**Table 2:** Level of ID in the final sample, separated by genetic syndrome

Syndrome (*N*)	Level of intellectual disability^a^						
	None (*N*)	Mild (*N*)	Moderate (*N*)	Severe (*N*)	Profound (*N*)	Unknown (*N*)	
AS *(13)*	1	0	0	5	3	4	
CdCS *(5)*	0	2	1	2	0	0	
CdLS *(16)*	0	1	6	7	1	1	
CSS *(7)*	0	0	4	2	0	1	
DS *(2)*	0	0	0	1	0	1	
FXS *(22)*	3	0	8	7	1	3	
JdVS *(7)*	0	0	3	3	0	1	
KBGS *(30)*	4	5	15	2	0	4	
KS *(19)*	2	2	4	7	3	1	
PKS *(5)*	0	0	0	1	3	1	
PWS *(9)*	3	1	3	2	0	0	
RTS *(23)*	1	2	6	9	1	4	
SAS *(29)*	1	3	8	9	5	3	
SMS *(14)*	0	1	5	5	0	3	
SYNGAP1 *(2)*	0	0	0	1	1	0	
TSC *(9)*	2	2	2	3	0	0	
WHS *(5)*	0	0	0	2	2	1	
WSS *(6)*	0	3	2	0	0	1	
Other *(15)*^*b*^	3	2	4	3	1	1	
Total *(238)*	19	24	71	71	21	30	

*AS *Angelman syndrome, *CdCS *Cri-du-Chat syndrome, *CdLS *Cornelia de Lange syndrome, *CSS *Coffin-Siris syndrome, *DS *Down syndrome, *FXS *Fragile X syndrome, *JdVS *Jansen-de Vries syndrome, *KBGS *KBG syndrome, *KS *Kleefstra syndrome, *PKS *Pallister-Killian syndrome, *PWS *Prader-Willi syndrome, *RTS *Rubinstein-Taybi syndrome, *SAS *SATB2-associated syndrome, *SMS *Smith-Magenis syndrome, *TSC *tuberous sclerosis complex, *WHS *Wolf-Hirschhorn syndrome, *WSS *Wiedemann-Steiner syndrome

^a^ Level of intellectual disability was reported by caregivers in the Background Questionnaire

^b^ The ’Other’ group includes 12 distinct syndromes. Details of these syndromes and syndrome-specific demographics are in Additional file 2

**Table 3:** Descriptive statistics for the Children's Sleep Habits Questionnaire, per syndrome

Syndrome (*N*)^a^	Median (IQR)								
	Bedtime Resistance (cutoff: 10.80)^b^	Sleep Onset Delay (cutoff: 2.30)^b^	Sleep Duration (cutoff: 5.30)^b^	Sleep Anxiety (cutoff: 7.80)^b^	Night Wakings (cutoff: 5.30)^b^	Parasomnias (cutoff: 10.60)^b^	SDB (cutoff: 4.50)^b^	Daytime Sleepiness (cutoff: 15.20)^b^	
**AS** *(13)*	6.50 *(4.00)*	1.00 *(1.75)*	5.00 *(3.00)*	5.00 *(3.00)*	6.00 *(2.75)*	10.00 *(2.50)*	3.00 *(1.00)*	10.00 *(3.50)*	
CdCS *(6)*	9.50 *(6.00)*	2.00 *(1.25)*	3.00 *(1.50)*	6.00 *(1.50)*	4.50 *(2.25)*	9.00 *(3.25)*	3.00 *(0.00)*	9.00 *(1.50)*	
**CdLS** *(19)*	8.00 *(5.00)*	2.00 *(1.75)*	3.50 *(2.75)*	5.00 *(2.75)*	4.00 *(1.00)*	9.50 *(4.50)*	3.00 *(1.50)*	9.50 *(4.75)*	
**CSS** *(10)*	7.00 *(4.00)*	1.00 *(0.00)*	3.00 *(1.00)*	6.00 *(2.00)*	3.00 *(3.00)*	10.00 *(5.00)*	4.00 *(2.00)*	11.00 *(2.00)*	
DS *(2)*	8.00 *(0.00)*	2.00 *(2.00)*	6.00 *(5.00)*	4.00 *(1.00)*	5.00 *(2.00)*	11.00 *(5.00)*	5.00 *(5.00)*	12.00 *(2.00)*	
**FXS** *(22)*	7.00 *(4.00)*	1.00 *(1.00)*	3.00 *(3.00)*	6.00 *(5.00)*	3.50 *(2.00)*	9.00 *(4.25)*	3.00 *(2.00)*	11.00 *(2.00)*	
JdVS *(7)*	8.00 *(6.00)*	1.00 *(1.00)*	4.00 *(1.00)*	7.00 *(2.00)*	4.00 *(3.00)*	11.00 *(2.00)*	4.00 *(2.25)*	11.00 *(4.00)*	
**KBGS ** *(39)*	8.00 *(5.00)*	1.00 *(1.00)*	4.00 *(3.00)*	5.00 *(3.00)*	5.00 *(3.00)*	9.00 *(4.00)*	3.00 *(1.00)*	12.00 *(3.00)*	
**KS** *(23)*	7.00 *(4.00)*	1.00 *(1.00)*	3.00 *(3.00)*	5.00 *(3.00)*	6.00 *(3.00)*	10.00 *(3.00)*	3.00 *(1.00)*	10.00 *(4.00)*	
PKS *(5)*	6.50 *(1.00)*	2.50 *(1.00)*	5.00 *(3.00)*	4.00 *(1.00)*	5.00 *(1.00)*	11.00 *(4.00)*	4.50 *(2.00)*	15.00 *(3.00)*	
PWS *(12)*	6.50 *(3.00)*	1.00 *(1.00)*	4.00 *(1.50)*	4.00 *(1.25)*	4.50 *(2.25)*	9.00 *(3.50)*	4.00 *(2.25)*	14.00 *(2.00)*	
**RTS ** *(26)*	6.00 *(2.25)*	1.00 *(1.00)*	3.00 *(2.00)*	4.00 *(2.00)*	4.00 *(4.00)*	10.00 *(4.00)*	4.00 *(2.25)*	11.00 *(4.25)*	
**SAS ** *(30)*	8.00 *(4.00)*	1.00 *(1.00)*	4.00 *(3.00)*	6.00 *(4.00)*	5.00 *(3.00)*	11.00 *(3.00)*	3.00 *(1.00)*	12.00 *(3.00)*	
**SMS** *(14)*	9.00 *(4.50)*	1.00 *(0.00)*	6.00 *(3.50)*	6.00 *(3.00)*	7.00 *(2.00)*	10.00 *(3.00)*	4.00 *(2.50)*	12.00 *(2.00)*	
SYNGAP1 *(3)*	10.00 *(7.00)*	2.00 *(2.00)*	4.00 *(2.00)*	6.00 *(4.00)*	7.00 *(3.00)*	10.00 *(0.25)*	3.00 *(3.00)*	13.00 *(5.00)*	
TSC *(9)*	7.00 *(2.00)*	2.00 *(1.00)*	3.00 *(2.00)*	5.00 *(2.00)*	5.00 *(2.00)*	9.00 *(2.00)*	3.00 *(2.00)*	14.00 *(4.00)*	
WHS *(5)*	9.50 *(6.00)*	1.00 *(0.00)*	5.00 *(4.00)*	6.00 *(5.00)*	5.50 *(4.00)*	9.00 *(2.00)*	3.00 *(1.00)*	11.00 *(6.00)*	
WSS *(8)*	8.50 *(8.50)*	2.00 *(1.75)*	3.50 *(5.00)*	7.00 *(3.50)*	4.50 *(4.75)*	10.00 *(3.25)*	3.50 *(4.50)*	10.50 *(4.50)*	

*AS *Angelman syndrome, *CdCS *Cri-du-Chat syndrome, *CdLS *Cornelia de Lange syndrome, *CSS *Coffin-Siris syndrome, *DS *Down syndrome, *FXS *Fragile X syndrome, *JdVS *Jansen-de Vries syndrome, *KBGS *KBG syndrome. KS: Kleefstra syndrome, *PKS *Pallister-Killian syndrome, *PWS *Prader-Willi syndrome, *RTS *Rubinstein-Taybi syndrome, *SAS *SATB2-associated syndrome, *SDB *sleep-disordered breathing, *SMS *Smith-Magenis syndrome, *TSC *tuberous sclerosis complex, *WHS *Wolf-Hirschhorn syndrome, *WSS *Wiedemann-Steiner syndrome

Syndromes highlighted in bold have *N* ≥ 10

^a^ N following pairwise deletion

^*b*^ Cut-off scores are 2 standard deviations above published means [62], scores exceeding these cut-offs indicate clinically-significant sleep problems [65–69]

To address the first aim, the proportion of children who scored above clinical thresholds for CSHQ subscale scores was compared between the syndrome groups and a previous TD sample [67]. The corresponding odds ratios are presented in Table 4. Although some syndrome-specific profiles of heightened sleep difficulties were demonstrated, several syndromes shared similar profiles (e.g. CSS, RTS, and KS, and SMS, SAS, and KBGS). Of particular note, a heightened risk of sleep-disordered breathing, parasomnias, and night awakenings was observed in most syndromes. No syndromes were characterised by a heightened risk of daytime sleepiness and only a few had a heightened risk of bedtime resistance (CdLS, KBGS, SAS, SMS, and WSS), sleep onset delay (AS, CdLS, KBGS, and WSS), sleep duration (AS, FXS, KBGS, SAS, and SMS), and sleep anxiety (WSS).

**Table 4:** Odds ratios for sleep difficulties measured by the Children's Sleep Habits Questionnaire

Syndrome (*N*)^a^	Odds ratios (95% CI)							
	Bedtime Resistance	Sleep Anxiety	Sleep Duration	Night Wakings	Parasomnias	SDB	Daytime Sleepiness	Sleep Onset Delay
AS *(13)*	3.77 *(0.62-* *23.06)*	1.40 *(0.27-* *7.25)*	**7.43** ***(2.04-*** ***27.00)***	**24.21** ***(5.50-*** ***106.54)***	**6.23** ***(1.21-*** ***31.91)***	5.09 *(0.76-* *33.91)*	0.37 *(0.04-* *3.05)*	**7.29** ***(1.65-*** ***32.15)***
CdLS *(19)*	**16.60** ***(4.23-*** ***65.19)***	0.96 *(0.19-* *4.82)*	3.33 *(0.96-* *4.82)*	2.59 *(0.44-* *15.38)*	**7.98** ***(1.89-*** ***33.65)***	**8.00** ***(1.61-*** ***39.64)***	0.26 *(0.03-* *2.11)*	**4.69** ***(1.12-*** ***19.61)***
CSS *(10)*	2.08 *(0.21-* *20.44)*	0.77 *(0.09-* *6.67)*	0.87 *(0.10-* *7.58)*	**7.78** ***(1.47-*** ***41.07)***	**17.29** ***(3.66-*** ***81.81)***	**23.33** ***(4.46-*** ***121.95)***	0.44 *(0.05-* *3.72)*	1.49 *(0.16-* *13.97)*
FXS *(22)*	5.76 *(0.92-* *23.61)*	5.92 *(0.49-* *17.02)*	**3.06** ***(2.33-*** ***9.74)***	**5.76** ***(5.91-*** ***23.61)***	**9.08** ***(1.63-*** ***34.67)***	**9.88** ***(1.16-*** ***43.44)***	0.20 *(0.05-* *1.61)*	0.75 *(0.81-* *6.71)*
KBGS *(39)*	**12.85** ***(3.80-*** ***43.45)***	2.00 *(0.69-* *5.77)*	**4.14** ***(1.53-*** ***11.22)***	**8.65** ***(2.49-*** ***30.04)***	**11.32** ***(3.32-*** ***38.54)***	2.71 *(0.52-* *14.14)*	0.43 *(0.12-* *1.58)*	**4.25** ***(1.25-*** ***14.51)***
KS *(23)*	4.37 *(1.00-* *19.05)*	2.14 *(0.65-* *7.03)*	3.06 *(0.96-* *9.74)*	**22.64** ***(6.20-*** ***82.61)***	**19.02** ***(5.21-*** ***69.42)***	**5.89** ***(1.22-*** ***28.55)***	0.42 *(0.09-* *1.99)*	2.46 *(0.54-* *11.16)*
PWS *(12)*	2.31 *(0.23-* *22.92)*	0.77 *(0.09-* *6.67)*	2.17 *(0.40-* *11.82)*	5.19 *(0.82-* *32.86)*	**8.89** ***(1.65-*** ***47.91)***	**18.67** ***(3.37-*** ***103.29)***	1.27 *(0.24-* *6.68)*	1.82 *(0.19-* *17.37)*
RTS *(26)*	4.15 *(0.95-* *18.04)*	0.70 *(0.14-* *3.43)*	2.28 *(0.69-* *7.59)*	**10.38** ***(2.79-*** ***38.61)***	**14.82** ***(4.08-*** ***53.87)***	**16.80** ***(4.07-*** ***69.33)***	0.63 *(0.17-* *2.39)*	2.34 *(0.52-* *10.60)*
SAS *(30)*	**10.38** ***(2.95-*** ***36.51)***	2.80 *(0.99-* *7.95)*	**4.33** ***(1.55-*** ***12.09)***	**12.01** ***(3.45-*** ***41.86)***	**27.13** ***(7.88-*** ***93.41)***	4.31 *(0.90-* *20.50)*	0.89 *(0.29-* *2.67)*	3.28 *(0.88-* *12.25)*
SMS *(14)*	**8.30** ***(1.79-*** ***38.46)***	2.10 *(0.50-* *8.83)*	**15.60** ***(4.28-*** ***56.83)***	**76.08** ***(15.00-*** ***385.89)***	**15.56** ***(3.62-*** ***66.91)***	**11.20** ***(2.19-*** ***57.41)***	0.34 *(0.04-* *2.80)*	1.09 *(0.12-* *10.03)*
TSC *(9)*	2.59 *(0.26-* *26.09)*	0.77 *(0.09-* *6.67)*	2.48 *(0.45-* *13.78)*	5.93 *(0.92-* *38.25)*	5.93 *(0.92-* *38.25)*	**14.00** ***(2.31-*** ***84.86)***	1.27 *(0.24-* *6.68)*	2.05 *(0.21-* *19.77)*
WSS *(8)*	**10.38** ***(1.87-*** ***57.42)***	**6.16** ***(1.42-*** ***26.81)***	4.33 *(0.92–20.38)*	**10.38** ***(1.87-*** ***57.42)***	**5.93** ***(1.05-*** ***45.73)***	**14.00** ***(2.31-*** ***84.86)***	0.44 *(0.05-* *3.72)*	**13.12** ***(2.66-*** ***64.66)***

*AS* Angelman syndrome, *CdLS *Cornelia de Lange syndrome, *CSS *Coffin-Siris syndrome, *FXS *Fragile X syndrome, *KBGS *KBG syndrome, *KS *Kleefstra syndrome, *PWS *Prader-Willi syndrome, *RTS *Rubinstein-Taybi syndrome, *SAS *SATB2-associated syndrome, *SMS *Smith-Magenis syndrome, *TSC *tuberous sclerosis complex, *WSS *Wiedemann-Steiner syndrome

Odds ratios are highlighted in bold where 95% CI did not cross 1.00 threshold

^a^ *N* following pairwise deletion

The same heightened sleep difficulties were observed following multiple imputation [see Additional file 6], however, additional syndromes had an increased likelihood of bedtime resistance (FXS, KS, and RTS), sleep duration (CdLS, KS, and WSS), night awakenings (CdLS, PWS, and TSC), parasomnias (TSC), and sleep-disordered breathing (AS, KGB, and SAS). As such, different profiles of heightened sleep difficulties were observed after pairwise deletion and multiple imputation. Following multiple imputation, all syndromes had a heightened risk of sleep-disordered breathing, parasomnias, and night awakenings, and different syndromes shared similar profiles of sleep difficulties (e.g. CSS, PWS, and TSC, and FXS, KS, SAS, and SMS).

**Fig. 2:**
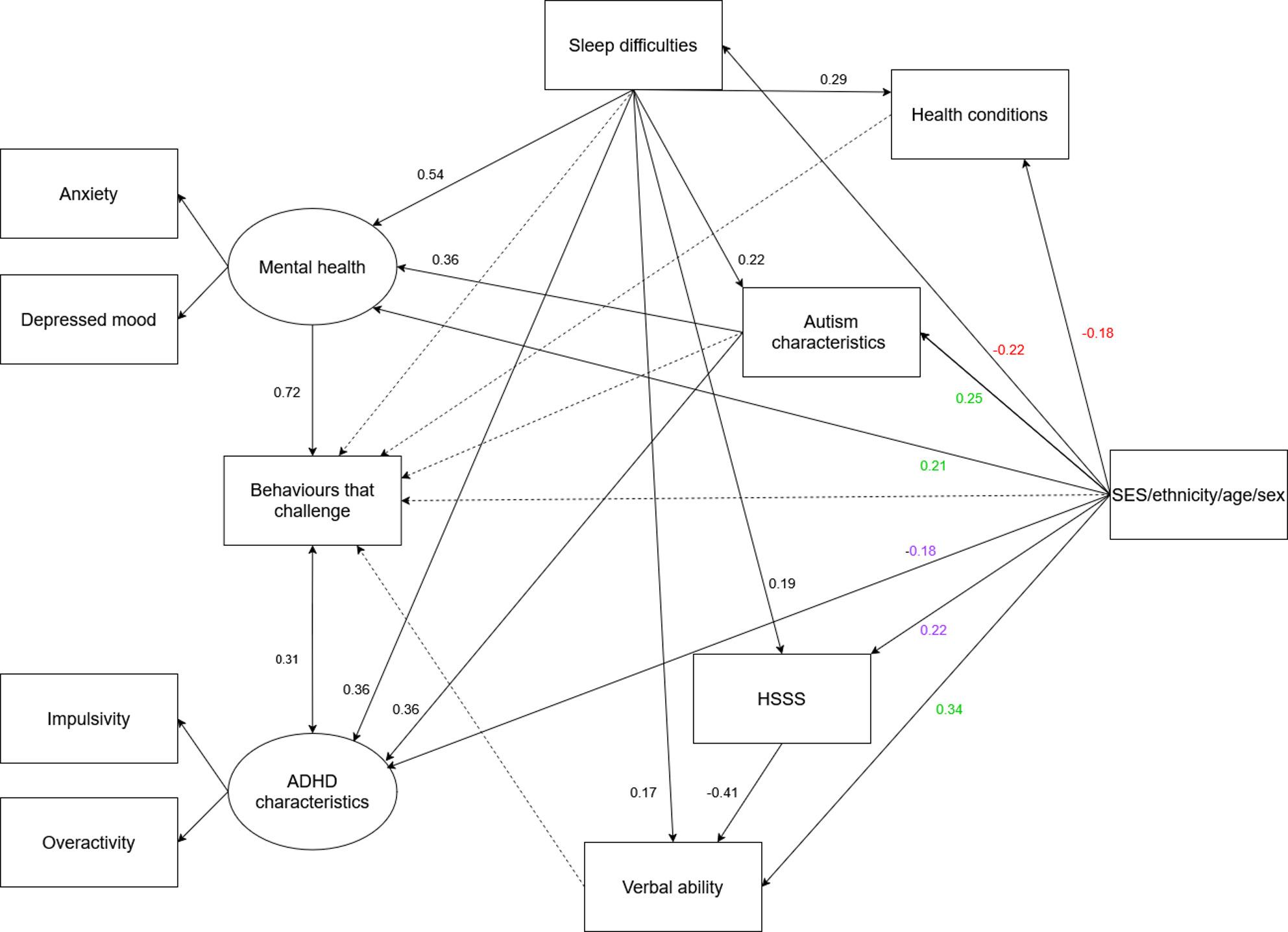
Path diagram summarising the standardised regression coefficients of the final structural equation model Note: ADHD: attention-deficit hyperactivity disorder. HSSS: Hague Seizure Severity Scale scores. SES: socioeconomic status. Values for statistically significant standardised regression coefficients are presented. Paths with solid lines were statistically significant (*p* < 0.05), paths with dashed lines were not statistically significant. Red coefficients represent socioeconomic status, green represent age, blue represent ethnicity, and purple represent sex. Although the arrows appear to visually indicate directionality, these represent regression analyses based on cross-sectional data and thus do not allow inferences of directionality

The second study aim was addressed via a SEM which examined the associations between sleep difficulties and broad clinical features whilst controlling for demographic characteristics and interactions between clinical features. The results of the SEM are presented in Fig. 2; Table 5,^7^. Of particular note, sleep difficulties were significantly, positively associated with mental health difficulties (β = 0.54), ADHD characteristics (β = 0.36), autism characteristics (β = 0.22), HSSS scores (β = 0.19), and the severity of physical health conditions (β = 0.29). Sleep difficulties were also greater for verbal children compared to non-verbal children (β = 0.17) and were not significantly associated with BtC. Moderate-to-large factor loadings and squared mean correlations were observed for the indicator variables (Table 6), supporting the construct validity of the latent variables. Additionally, although the pre-defined fitness indices’ criteria were breached in some cases, the combined results broadly indicate a satisfactory fit: χ^2^ = 76.42, df = 34, *p* < 0.001; CFI = 0.92; TLI = 0.90; SRMSR = 0.09; RMSEA = 0.08 [90% CI 0.054–0.099].

**Table 5:** Regression coefficients from the structural equation model

Dependent variable	Predictor variable	B (95% CI)	β	SE	*p*	SMC
BtC	Sleep difficulties	-0.09 (-0.65; 0.46)	-0.08	0.28	0.75	0.34
	Verbal ability	0.05 (-0.14; 0.24)	0.06	0.10	0.63	
	Autism characteristics	-0.10 (-0.47; 0.26)	-0.10	0.19	0.58	
	**Mental health**	**0.76 (0.05; 1.48)**	**0.72**	**0.36**	**0.04**	
	Health conditions	0.05 (-0.19; 0.29)	0.05	0.12	0.66	
	SES	0.10 (-0.18; 0.38)	0.08	0.14	0.49	
	Age	-0.18 (-0.43; 0.07)	-0.18	0.13	0.17	
	Ethnicity	-0.18 (-0.79; 0.42)	-0.06	0.31	0.56	
	Sex	-0.08 (-0.50; 0.35)	-0.04	0.22	0.73	
Mental health	**Sleep difficulties**	**0.56 (0.38; 0.73)**	**0.54**	**0.09**	**< 0.001**	0.55
	**Autism characteristics**	**0.35 (0.17; 0.53)**	**0.36**	**0.09**	**< 0.001**	
	SES	0.10 (-0.07; 0.28)	0.09	0.09	0.24	
	**Age**	**0.19 (0.03; 0.35)**	**0.21**	**0.08**	**0.02**	
	Ethnicity	-0.03 (-0.46; 0.40)	-0.01	0.22	0.88	
	Sex	0.04 (-0.25; 0.34)	0.02	0.15	0.78	
ADHD characteristics	**Sleep difficulties**	**0.32 (0.18; 0.46)**	**0.36**	**0.07**	**< 0.001**	0.42
	**Autism characteristics**	**0.30 (0.16; 0.45)**	**0.36**	**0.08**	**< 0.001**	
	SES	0.06 (-0.08; 0.20)	0.06	0.07	0.39	
	Age	0.11 (-0.01; 0.22)	0.13	0.06	0.07	
	Ethnicity	-0.10 (-0.27; 0.47)	0.04	0.19	0.59	
	**Sex**	**-0.29 (-0.52; -0.07)**	**-0.18**	**0.12**	**0.01**	
Autism characteristics	**Sleep difficulties**	**0.23 (0.09; 0.37)**	**0.22**	**0.07**	**0.001**	0.19
	SES	-0.11 (-0.29; 0.07)	-0.09	0.09	0.23	
	**Age**	**0.25 (0.12; 0.39)**	**0.26**	**0.07**	**< 0.001**	
	Ethnicity	-0.03 (-0.40; 0.47)	0.01	0.22	0.88	
	Sex	-0.02 (-0.30; 0.26)	-0.01	0.14	0.89	
Verbal ability	**Sleep difficulties**	**0.21 (0.04; 0.39)**	**0.17**	**0.09**	**0.02**	0.34
	**HSSS scores**	**-0.47 (-0.70; -0.24)**	**-0.41**	**0.12**	**< 0.001**	
	SES	0.04 (-0.22; 0.30)	0.03	0.14	0.77	
	**Age**	**0.38 (0.18; 0.58)**	**0.34**	**0.10**	**< 0.001**	
	Ethnicity	-0.17 (-0.76; 0.42)	-0.05	0.30	0.58	
	Sex	-0.07 (-0.50; 0.36)	-0.03	0.22	0.75	
HSSS scores	**Sleep difficulties**	**0.21 (0.05; 0.38)**	**0.19**	**0.09**	**0.01**	0.10
	SES	0.09 (-0.19; 0.36)	0.07	0.14	0.53	
	Age	0.11 (-0.11; 0.32)	0.11	0.11	0.32	
	Ethnicity	0.11 (-0.51; 0.73)	0.04	0.32	0.74	
	**Sex**	**0.45 (0.01; 0.89)**	**0.22**	**0.22**	**0.04**	
Health conditions	**Sleep difficulties**	**0.30 (0.20; 0.39)**	**0.29**	**0.05**	**< 0.001**	0.15
	**SES**	**-0.18 (-0.34; -0.02)**	**-0.16**	**0.14**	**0.03**	
	Age	-0.02 (-0.15; 0.12)	-0.02	0.07	0.83	
	Ethnicity	0.08 (-0.32; 0.48)	0.03	0.20	0.69	
	Sex	0.07 (-0.20; 0.35)	0.04	0.14	0.61	
Sleep difficulties	**SES**	**-0.25 (-0.39; -0.10)**	**-0.22**	**0.08**	**0.001**	0.07
	Age	-0.04 (-0.53; 0.26)	-0.05	0.07	0.52	
	Ethnicity	-0.13 (-0.18; 0.09)	-0.05	0.20	0.51	
	Sex	-0.18 (-0.46; 0.10)	-0.10	0.14	0.20	

*ADHD *attention-deficit hyperactivity disorder, *HSSS *Hague Seizure Severity Scale, *SE *standard error, *SMC *squared multiple correlation. *B *unstandardised regression coefficients, *β *standardised regression coefficients

*SE* and *p* corresponds to unstandardised regression coefficients. Significant results (*p* < 0.05) are highlighted in bold

**Table 6:** Results from measurement component of the structural equation model

Latent variable	Indicator variable	SMC	Unstd loading	Std loading	SE	*p*
Mental health	ClASP-ID anxiety	0.86	1.00	0.93	-	-
	MIPQ total	0.35	-0.60	-0.59	0.08	< 0.001
ADHD characteristics	TAQ impulsivity	0.73	1.00	0.85	-	-
	TAQ overactivity	0.64	0.95	0.80	0.13	< 0.001

*CIASP-ID *Clinical Anxiety Screen for people with Severe to Profound Intellectual Disability, *MIPQ *Mood Interest and Pleasure Questionnaire, *SE *standard error, *SMC *squared mean correlations, *Std *standardised, *TAQ *The Activity Questionnaire, *Unstd *unstandardised

*SE* corresponds to the unstandardised loadings

Most of the significant associations were replicated following multiple imputation [see Additional file 6]. Only the associations between sleep difficulties and verbal ability, and HSSS scores and sex, were no longer significant. A significant positive association also emerged between ADHD characteristics and age, suggesting ADHD characteristics increased with age. Similar model fit was also evidenced after multiple imputation.

In correspondence with the SEM, the logistic regressions for BtC topographies revealed no evidence of associations between sleep difficulties and BtC [see Additional file 7]. These results were replicated following multiple imputation [see Additional file 6].

The final study aim was addressed by the SEM which examined the associations between sleep difficulties and demographic characteristics. Sleep difficulties were significantly negatively associated with SES (β=-0.22) but were not associated with other demographic characteristics (Table 5). The same pattern of significance across associations was retained following multiple imputation [see Additional file 6]. As indicated by the SMC, the combined demographic characteristics explained 7% of the variance in sleep difficulties.

## Discussion

This study aimed to (i) describe profiles of sleep difficulties amongst children with rare genetic syndromes, including syndromes with little-to-no existing description, (ii) advance our understanding of the associations between sleep difficulties and broad clinical features in rare genetic syndromes, and (iii) delineate the associations between sleep difficulties and demographic characteristics in rare genetic syndromes. The findings are bolstered by a large sample, facilitated by the inclusion of many rare genetic syndromes. The use of multiple imputation also determined the robustness of results to data loss, facilitating rigorous inferences. Additionally, the observed associations between sleep difficulties and broad clinical features were enhanced by controlling the effects of demographic characteristics and interactions between clinical features.

To address the first study aim, the proportion of children that scored above the CSHQ subscale clinical thresholds was compared between the syndrome groups and the TD comparison group. Different sleep difficulties were observed for several syndromes after pairwise deletion and multiple imputation, indicating bias attributable to the missing data. Therefore, the results following multiple imputation, which are more robust to missing data [105], are discussed in detail. The findings broadly demonstrate a heightened risk of caregiver-defined sleep difficulties in children with rare genetic syndromes, corroborating previous meta-analyses and reviews [1, 106, 107]. Notably, the results are the first to describe profiles of sleep difficulties within many rare genetic syndromes (*N* = 12) using the same measure and comparison group, offering new insights into the ubiquity of specific sleep difficulties across syndromes. Although some syndrome-specific profiles of sleep difficulties were observed (e.g. AS, CdLS, KBGS, WSS), several syndromes shared similar profiles of sleep difficulties (e.g. CSS, PWS, and TSC). Indeed, five of the eight sleep difficulties assessed by the CSHQ were heightened across most syndromes (night awakenings, parasomnias, sleep-disordered breathing, bedtime resistance, and sleep duration). The ubiquity of specific sleep difficulties may be partially driven by the low rates observed in the TD comparator [67], although these low rates are comparable to those of previous large cohort studies of TD children [108–110]. Similar to the current results, previous research has identified recurrent sleep difficulties across different rare genetic syndromes. For example, reviews have described sleep-disordered breathing and/or night awakenings in CdLS, DS, RST, AS, FXS, PWS, and SMS [106, 107, 111] and meta-analytic evidence has highlighted the commonality of insomnia-related difficulties, sleep-disordered breathing, and daytime sleepiness across genetic syndromes [1]. The recurrence of these specific sleep difficulties across syndromes further characterises the pervasiveness of poor sleep in rare genetic syndromes and highlights the need for clinical sleep services *across* syndromes.

Amongst the examined syndromes were those with little-to-no prior description of sleep difficulties, particularly for samples of children. Indeed, this study is the first to describe specific sleep difficulties in children with CSS, KBGS, and KS using a standardised sleep questionnaire. Additionally, this study has progressed the descriptions of sleep difficulties experienced by children with WSS and SAS which had rarely been assessed with standardised sleep measures. With the exception of parasomnias and sleep duration, the sleep difficulties in the WSS group correspond to previous results [31]. For the SAS group, the lower risk of daytime sleepiness and sleep onset delay, and heightened risk of parasomnias and night wakings, corroborates previous findings [32, 112]. In contrast, the heightened risk of sleep-disordered breathing in the current SAS sample conflicts with previous findings [32, 112], possibly due to the use of different sleep questionnaires. Overall, the current findings advance previously scarce descriptions of sleep difficulties experienced by children with CSS, KBGS, KS, WSS, and SAS. The findings highlight the potential relevance of sleep difficulties within the behavioural phenotype of each syndrome and demonstrate the need for further research into the presentation and impact of sleep difficulties within these syndromes.

Of particular note, no syndromes had an elevated risk of daytime sleepiness compared to the TD group, conflicting with robust reports of heightened daytime sleepiness in syndromes such as AS, WS, PWS, SMS, and TSC [1, 113]. This finding can be attributed to the prevalence of low daytime sleepiness scores in the current sample, as opposed to inflated scores in the TD comparator [67]. These low scores may be explained as an artefact of the CSHQ. Indeed, alternate measures (such as the Modified Simonds & Parraga Sleep Questionnaire, Modified/Unmodified Epworth Sleepiness Scale, Pediatric Sleep Questionnaire, study-specific questionnaires, and daytime periods of sleep detected by polysomnography) have consistently indicated elevated daytime sleepiness in syndromes such as AS [11, 114], WS [12], PWS [23, 115, 116], and SMS [59, 117]. In contrast, studies of these syndromes that rely on the CSHQ have not found elevated daytime sleepiness scores relative to TD peers [58, 63, 118, 119]. The low daytime sleepiness ratings may also be explained by the young median age of several syndrome groups (e.g. KBGS, KS, and TSC). Younger children can express daytime sleepiness less overtly and instead exhibit non-somnolent behaviour, such as overactivity or BtC, possibly affecting caregivers’ daytime sleepiness ratings [120, 121]. Overall, daytime sleepiness is challenging to measure in individuals with intellectual disabilities given that this is, in-part, an internal subjective state [122] that cannot be easily self-reported due to delays in receptive language, expressive language, and introspection abilities. Nevertheless, the CSHQ is one of the most psychometrically tested sleep questionnaires available for children with rare genetic syndromes and the subscales broadly demonstrate good convergence with sleep difficulties/parameters recorded via sleep diaries and medical history forms [123]. Future studies should further investigate the measurement properties of the CSHQ, particularly the daytime sleepiness subscale, in rare genetic syndromes and consider utilising multi-method assessments of daytime sleepiness.

In addressing the second aim, this study was the first to describe the associations between sleep difficulties and other clinical features in children with rare genetic syndromes, after controlling for demographic characteristics and interactions between clinical features. With a few exceptions, the results were similar following listwise deletion and multiple imputation, indicating minimal bias attributable to the missing data. The analysis demonstrated that sleep difficulties were positively associated with mental health difficulties, ADHD-related characteristics, autism-related characteristics, verbal ability, severity of physical health conditions, and HSSS scores. These findings offer greater rigour, yet nonetheless replicate, previous positive bivariate associations between sleep difficulties and each of these clinical features (e.g. [2, 7, 11, 13, 15–17, 124]). Crucially, these results demonstrate that sleep difficulties are relevant to a variety of clinical outcomes for children with rare genetic syndromes, even after controlling for other predictors. As such, the observed associations should be explored further to determine whether sleep is an effective modifiable target to improve children’s outcomes. With respect to clinical implications, the current findings may enhance detection of sleep difficulties in children with rare genetic syndromes. More specifically, the findings outline several clinical features that are associated with sleep difficulties and may be more readily observed by caregivers and clinicians (e.g. mental health difficulties or physical health conditions). Caregivers and/or clinicians that observe these clinical features may be prompted to screen for sleep difficulties or refer children to a sleep clinician. This is especially relevant for non-sleep specialists who typically have limited sleep training and may not consistently or rigorously screen for sleep difficulties [125].

Unexpectedly, a null association was observed between sleep difficulties and BtC in the SEM and topography-specific logistic regression analyses, conflicting with positive associations throughout past studies [2–4, 6, 7, 124]. In contrast, mental health difficulties and ADHD-associated characteristics (overactivity and impulsivity) were positively associated with BtC, corresponding to well-established associations [37, 80, 92, 94, 126]. Studies of rare genetic syndromes have rarely controlled for mental health difficulties and/or ADHD-associated characteristics when estimating associations between sleep and BtC, limiting available comparisons. In conflict with the current findings, one study of AS found that sleep difficulties were significantly associated with BtC but not with mental health difficulties [7]. In further contrast with the current findings, studies of autism have demonstrated that sleep difficulties are independently, positively associated with BtC, mental health difficulties, and overactivity/impulsivity [5, 127–129]. These discordant findings may be explained as BtC was analysed as a binary variable in the present study, limiting the information available for analysis and possibly obscuring the cumulative effect of sleep difficulties on the severity of BtC. Alternatively, given the strong indirect paths between sleep difficulties and BtC, via mental health difficulties and ADHD-associated characteristics (see Fig. 2), it is possible the direct effect of sleep difficulties on BtC is mediated by mental health difficulties and/or overactivity/impulsivity. To extend the current findings, future studies should investigate the mechanisms that link sleep difficulties with BtC and mental health difficulties in individuals with rare genetic syndromes. Future studies should also strive to quantify sleep, BtC, and mental health difficulties using continuous variables and other direct assessment measures in addition to caregiver-report.

To address the final study aim, associations between sleep difficulties and various demographic characteristics were examined across the combined syndromes. Almost identical findings were obtained after listwise deletion and multiple imputation, suggesting that missing data did not substantially bias the results. Only SES was significantly associated with sleep difficulties, with greater sleep difficulties observed for children with lower SES compared to those with higher SES. This finding is critical as the interactions between SES and sleep difficulties have only been examined in one previous study of rare genetic syndromes [8], which found a null association between caregiver income and CSHQ total scores. The conflicting results may be attributed to differences in the measurement of SES, however, this requires further investigation. Nevertheless, the negative association between sleep difficulties and SES replicates well-established trends from TD [57, 130] and autistic [131, 132] samples, suggesting that environmental factors that are associated with poor sleep in these populations may generalise to rare genetic syndromes. Indeed, environmental correlates of poor sleep in the general population, such as SES, family conflict [133], school/work schedules [134] and daylight savings [135], have rarely been examined in rare genetic syndromes and warrant further investigation. However, given the limited variance in sleep difficulties that was explained by demographic characteristics (7%), the clinical significance of SES-sleep difficulty associations in biologically determined rare syndromes should not be overstated. Nevertheless, acknowledging environmental determinants of sleep difficulties is integral to mitigate against the emergence of sleep difficulties, improve the early identification of sleep difficulties, and inform the allocation of clinical sleep services. Future research should further examine the relevance of environmental factors, such as SES, to sleep difficulties in rare genetic syndromes and specifically address the directionality of this association. Regarding other demographic characteristics, the lack of association between sleep difficulties and age corresponds to previous null associations throughout studies of rare genetic syndromes [11, 15, 114, 136–138]. The lack of evidence supporting the remission of sleep difficulties highlights the need for further longitudinal investigation to confirm the association, or lack thereof, between age and specific sleep difficulties in children with sleep difficulties. Notably, this study examined chronological age rather than developmental age. Rigorous assessment of developmental ability was not possible as there is not currently a dedicated informant-report measure of developmental ability for people with intellectual disabilities. Nevertheless, chronological age and developmental age can notably differ amongst individuals with intellectual disabilities, potentially limiting the representativeness of chronological age of individuals’ developmental status. Therefore, in addition to chronological age, future studies should directly explore the associations between developmental age and sleep difficulties in children with rare genetic syndromes associated with intellectual disability. A null association was also demonstrated between sleep difficulties and sex, aligning with previous research in rare genetic syndromes [8, 17, 139, 140]. Similarly, ethnicity was not associated with sleep difficulties. No other studies of rare genetic syndromes have yet explored the associations between ethnicity and sleep difficulties. However, the null association should be interpreted carefully as the current sample was predominantly white, limiting the representativeness of other ethnic groups.

### Study limitations

When interpreting the current findings, several limitations should be acknowledged. Firstly, given the cross-sectional nature of the current study and preceding studies (e.g. [2, 11, 124]), the causal directionality of many associations between sleep difficulties and clinical features remains unclear. To examine *how* sleep and clinical outcomes are associated, studies should employ methodologies capable of directional inferences (such as longitudinal or intervention studies). Secondly, the data were entirely drawn from caregiver-completed questionnaires which are vulnerable to subjective biases [141, 142] and do not capture temporal variation in sleep [59, 143] or in clinical features, such as overactivity and impulsivity [144–146] and anxiety [147]. However, the convenience of questionnaires facilitated data collection for a large sample which enabled data-intensive analyses. Thirdly, the syndrome samples were generally disproportional, potentially skewing the SEM results towards those syndromes with the largest samples. Indeed, five syndrome groups constituted 123/238 (52%) children in the final sample. Nevertheless, no single syndrome dominated the sample, ensuring the results retained cross-syndrome generalisability. Another limitation was that the TD comparator group was Italian whereas the current sample were predominantly British and North American, potentially introducing cross-cultural biases to the comparisons [148, 149]. However, the only other published TD comparators were from mainland China which has relatively greater cultural differences in sleep [150] and caregivers’ perceptions of sleep [151]. Additionally, the current sample was skewed towards higher SES households, reducing the representativeness of the results for individuals from lower SES backgrounds. Future studies of rare genetic syndromes should aim to increase the accessibility of research to lower income households. Additionally, although this study advanced the modelling of interactions between sleep difficulties and clinical features in children with rare genetic syndromes, not all biological determinants of sleep difficulties were investigated (such as craniofacial differences or muscular hypotonia). Future studies that address the interactions between sleep difficulties and key clinical features in rare genetic syndromes could utilise patient registry data to rigorously assess the purported biological pathways. Finally, the associations between sleep difficulties and clinical features were examined using total scores, composite scores, and latent variables. The non-specific nature of these data limited the precision of the findings and arguably precluded insights into the mechanisms that link sleep difficulties with clinical features. However, these broad variables were necessitated to streamline the data-intensive, multivariate analyses.

## Conclusion

This study described profiles of sleep difficulties across rare genetic syndromes, examined the relevance of sleep difficulties to children’s clinical outcomes, and explored the impact of demographic characteristics on sleep difficulties. Sleep difficulties were heightened across all syndromes, compared to TD peers, and were associated with several clinical features after controlling for other predictors. Of the demographic characteristics, only SES was associated with sleep difficulties. These findings warrant further research into (i) the profiles and impacts of sleep difficulties syndromes with little existing sleep research, (ii) the causal mechanisms linking sleep difficulties and clinical features, and (iii) the interactions between demographic characteristics, particularly SES and age, and sleep difficulties.

## Supplementary Information

Additional file 1: Number of caregivers recruited from each country.

Additional file 2: Demographic characteristics of children with ‘other’ rare genetic syndromes.

Additional file 3: Variables from the background questionnaire and response options.

Additional file 4: Number of children with confirmed syndrome diagnoses, per syndrome.

Additional file 5: Assessment of missing data and multiple imputation procedure.

Additional file 6: Results following multiple imputation.

Additional file 7: Results of the logistic regressions examining different topographies of behaviours that challenge.

## Data Availability

The datasets analysed in the current study are available from the corresponding author, upon reasonable request.

## Footnotes

1 The second aim outlined in the pre-registration, and the corresponding analyses, were omitted due to the small samples of most syndromes which precluded sufficiently powered within-syndrome correlations and between-syndrome comparisons, especially following *p*-value correction.

2 For the full list of included measures in the BEOND study please refer to the BEOND study pre-registration: 10.17605/OSF.IO/N89X7.

3 The internal consistency of the CBQ subscales may be limited as the individual items, and corresponding behaviours, that constitute the subscales may not be inherentlyassociated. For example, although self-injury may occur frequently (equating to a high rating), individual instances of self-injury may be short in duration (equating to a low rating).

4 Few studies report the percentage of TD children that score above CSHQ cut-off scores (Pilotto et al.,[67]; Wang et al., [﻿]). Pilotto et al. [67] was chosen for the current comparisons as the children were recruited from a Western culture, as done for the current study, and the rates of sleep difficulties were similar to those in large TD cohort studies (Bixler et al., [108]; Hochadel et al., [109]; Laberge et al., [110]). Additionally, the sample reported by Pilotto et al. [67] was similar in age(2-16 years) to the current sample (1-18 years), limiting the effects of age on between-group comparisons. Conversely, Wang et al. [69] recruited a mainland Chinese sample which may have introduced cross-cultural biases to the comparisons (Jeon et al., [150]; Mindell et al., 2013; Sadeh et al., [151]).

5 These comparisons differed from those specified in the pre-registration, as insufficient data were collected for TD children and children with non-syndromic ID to support the planned comparisons.

6 The ‘ability’ latent variable described in the pre-registration was incalculable as the corresponding items were binary (verbal ability) and categorical (Wessex self-help). The ‘externalising’ latent variable specified in the pre-registration was not included as this was zero inflated and precluded model fit.

7 In addition to the sleep-daytime feature associations, the SEM also demonstrated that (i) autism characteristics were positively associated with ADHD characteristics and mental health difficulties, (ii) mental health difficulties were positively associated with BtC, and (iii) verbal ability was negatively associated with epilepsy severity. A positive, significant covariance was also observed between ADHD characteristics and BtC (*r*=0.31, *p*<0.001). Mental health difficulties, autism characteristics, and verbal ability were also positively associated with age. Compared to males, females had significantly greater epilepsy severity and lower ADHD characteristics.

## Abbreviations

ADHD: Attention-Deficit Hyperactivity Disorder
AS: Angelman Syndrome
BtC: Behaviours that Challenge
CdCS: Cri du Chat Syndrome
CdLS: Cornelia De Lange Syndrome
CSHQ: Children’s Sleep Habits Questionnaire
CSS: Coffin-Siris Syndrome
DS: Down Syndrome
FXS: Fragile X Syndrome
JdVS: Jansen-de Vries Syndrome
KBGS: KBG Syndrome
KS: Kleefstra Syndrome
PKS: Pallister-Killian Syndrome
PWS: Prader-Willi Syndrome
RS: Rett Syndrome
RTS: Rubinstein-Taybi Syndrome
SAS: SATB2-Associated Syndrome
SEM: Structural Equation Modelling
SES: Socioeconomic Status
SMS: Smith-Magenis Syndrome
TAQ: The Activity Questionnaire
TD: Typically-Developing
TSC: Tuberous Sclerosis Complex
WHS: Wolf-Hirschhorn Syndrome
WS: Williams Syndrome
WSS: Wiedemann-Steiner Syndrome
