# Supplementary material for: The interactions between sleep difficulties and clinical features in children with rare genetic syndromes: a structural equation modelling approach
Source: BMC Pediatr. 2026 Jun 5;26:740. doi: 10.1186/s12887-026-07099-5 (PMC13471272; doi:10.1186/s12887-026-07099-5)
Supplement: Supplementary file 1 [file 12887_2026_7099_MOESM1_ESM.pdf]

Additional file 1: Number of caregivers recruited from each country

Country	Number of caregivers in the final sample
United Kingdom	121
United States of America	65
Argentina	1
Australia	11
Canada	4
Finland	1
France	1
Germany	4
India	1
Indonesia	1
Ireland	3
Italy	3
Netherlands	2
Poland	3
Portugal	2
Russia	1
Slovenia	3
South Africa	3
Spain	8
