# Supplementary material for: The interactions between sleep difficulties and clinical features in children with rare genetic syndromes: a structural equation modelling approach
Source: BMC Pediatr. 2026 Jun 5;26:740. doi: 10.1186/s12887-026-07099-5 (PMC13471272; doi:10.1186/s12887-026-07099-5)
Supplement: Supplementary file 2 [file 12887_2026_7099_MOESM2_ESM.pdf]

**Additional file 2: Demographic characteristics of children with 'other' rare genetic syndromes**

| Syndrome *(N)* | Mean Age (Y) | Male/ female (N) | Ethnicity | | | | | Socioeconomic status | | | |
|---|---|---|---|---|---|---|---|---|---|---|---|
| | | | Wh (N) | Mi (N) | As (N) | Bl (N) | Oth (N) | <NA (N) | ~NA (N) | >NA (N) | ND (N) |
| 19p13.13 deletion (1) | 8.18 | 7/8 | 15 | 0 | 0 | 0 | 0 | 3 | 3 | 8 | 1 |
| 10q22-10q23 del (1) | | | | | | | | | | | |
| 15q11.2q13.1 & 17p13.3 mdup (1) | | | | | | | | | | | |
| 16p11.2 dup (2) | | | | | | | | | | | |
| 1q21.1q21.2 (2) | | | | | | | | | | | |
| 1q42 dup (1) | | | | | | | | | | | |
| 22q11.2 del (1) | | | | | | | | | | | |
| 2p16.3 mdel (1) | | | | | | | | | | | |
| 8p inv dup/del (1) | | | | | | | | | | | |
| RSS (2) | | | | | | | | | | | |
| Shank 2 del (1) | | | | | | | | | | | |
| WS (1) | | | | | | | | | | | |

*Note.* As: Asian or Asian British. Bl: Black, African, Caribbean, or Black British. ID: intellectual disability. del: deletion. dup: duplication. GS: Glass syndrome. inv: inverted. mdel: microdeletion. mdup: microduplication. Mi: Mixed ethnicity or multiple ethnic groups. NA: national average household income. ND: Not disclosed. Oth: Other. RSS: Russell Silver syndrome. SES: socioeconomic status. Wh: White. WS: Weaver syndrome. Y: years.

| Syndrome *(N)* | Level of intellectual disability | | | | | |
|---|---|---|---|---|---|---|
| | None (N) | Mild (N) | Mod (N) | Sev (N) | Prof (N) | Unk (N) |
| 19p13.13 deletion (1) | 5 | 2 | 3 | 2 | 1 | 2 |
| 10q22-10q23 del (1) | | | | | | |
| 15q11.2q13.1 & 17p13.3 mdup (1) | | | | | | |
| 16p11.2 dup (2) | | | | | | |
| 1q21.1q21.2 (2) | | | | | | |
| 1q42 dup (1) | | | | | | |
| 22q11.2 del (1) | | | | | | |
| 2p16.3 mdel (1) | | | | | | |
| 8p inv dup/del (1) | | | | | | |
| RSS (2) | | | | | | |
| Shank 2 del (1) | | | | | | |
| WS (1) | | | | | | |

*Note.* del: deletion. dup: duplication. GS: Glass syndrome. inv: inverted. mdel: microdeletion. mdup: microduplication. Mild: Mild intellectual disability. Mod: Moderate intellectual disability. Sev: Severe intellectual disability. Prof: Profound intellectual disability. Unk: Unknown level of intellectual disability. RSS: Russell Silver syndrome. WS: Weaver syndrome.
