# Supplementary material for: The interactions between sleep difficulties and clinical features in children with rare genetic syndromes: a structural equation modelling approach
Source: BMC Pediatr. 2026 Jun 5;26:740. doi: 10.1186/s12887-026-07099-5 (PMC13471272; doi:10.1186/s12887-026-07099-5)
Supplement: Supplementary file 3 [file 12887_2026_7099_MOESM3_ESM.pdf]

Additional file 3: Variables from the background questionnaire and response options

Measured variable	Response options
Sex	Male<br>Female<br>Prefer not to say<br>Prefer to self-describe as (free-text response)
Ethnicity	White<br>Mixed or multiple ethnic groups<br>Asian or Asian British<br>Black, African, Caribbean, or Black British<br>Other (free-text response)
Socioeconomic status	Below national average<br>Similar to national average<br>Above national average<br>Prefer not to say
Verbal ability	≥30 signs/words in vocabulary<br><30 signs/words in vocabulary
Level of intellectual disability	Mild<br>Moderate<br>Severe<br>Profound<br>Unknown<br>Other (free-text response)
