# Supplementary material for: The interactions between sleep difficulties and clinical features in children with rare genetic syndromes: a structural equation modelling approach
Source: BMC Pediatr. 2026 Jun 5;26:740. doi: 10.1186/s12887-026-07099-5 (PMC13471272; doi:10.1186/s12887-026-07099-5)
Supplement: Supplementary file 4 [file 12887_2026_7099_MOESM4_ESM.pdf]

Additional file 4: Number of children with confirmed syndrome diagnoses, per syndrome

Syndrome	Number with genetic confirmation	Total number of children
Angelman syndrome	5	13
Cri du Chat syndrome	2	5
Cornelia de Lange syndrome	3	16
Coffin-Siris syndrome	4	7
Down syndrome	1	2
Fragile X syndrome	7	22
Jansen de Vries syndrome	4	7
KBG syndrome	12	30
Kleefstra syndrome	11	19
Pallister-Killian syndrome	2	5
Prader-Willi syndrome	4	9
Rubinstein-Taybi syndrome	8	23
SATB2-associated syndrome	13	29
Smith-Magenis syndrome	4	14
SYNGAP1	1	2
Wolf-Hirschhorn syndrome	1	5
Wiedemann-Steiner syndrome	2	6
Other	8	15
