# Supplementary material for: The interactions between sleep difficulties and clinical features in children with rare genetic syndromes: a structural equation modelling approach
Source: BMC Pediatr. 2026 Jun 5;26:740. doi: 10.1186/s12887-026-07099-5 (PMC13471272; doi:10.1186/s12887-026-07099-5)
Supplement: Supplementary file 5 [file 12887_2026_7099_MOESM5_ESM.pdf]

Additional file 5: Assessment of missing data and multiple imputation procedure

Across the analysed variables, the total proportion of missing data was 16%. Rates of missingness in individual variables ranged from 0% (sex and age) to 29% (ClASP-ID anxiety subscale). Missingness rates for all variables are outlined in Figure 1. A sample of 238 was available following listwise deletion from an initial sample of 340.

Missingness was examined via diagnostic plots, produced with the *ggplot2* (Wickham, 2016) and *naniar* (Tierney & Cook, 2023) R packages. Missingness was not notably influenced by syndrome, SES, degree of adaptive functioning, caregiver education, child age, caregiver age, or country (see Figures 2-9). However, order effects appeared to affect missingness as consistently lower rates of missingness were observed for background questionnaire variables, presented first in the survey, relative to variables of other questionnaires. Since the questionnaires were counterbalanced following the background questionnaire, the effect of questionnaire order on missingness was considered relatively well controlled. Indeed, rates of missingness were predominantly consistent across the remaining questionnaires. Under this assumption, the data were considered missing at random (MAR), not missing *completely* at random (MCAR). Indeed, Little's missing MCAR was significant (χ^2=714.0(640), *p*=0.02), indicating the data were not MCAR. Under the assumption of MAR, the data were suitable for multiple imputation (Bhaskaran & Smeeth, 2014).

Missing data were replaced via multiple imputation using the *mice* (van Buuren & Groothuis-Oudshoorn, 2011), *miceadds* (Robitzsch & Grund, 2024), and *countimp* packages (Kleinke & Reinecke, 2019) in R studio. Multiple imputation can reduce bias in estimates and increase precision relative to listwise deletion (Madley-Dowd et al., 2019; Woods et al., 2024). Alongside sleep and daytime clinical feature variables, ethnicity (missingness: 1%) and SES (missingness: 2%) were imputed. Imputing demographic characteristics such as these is an established practice (Silva et al., 2019) and, given the small percentage of missing data, the risk of misrepresenting ethnic groups and perpetuating the underrepresentation of low-income households/ethnic minorities in research was considered minimal (Lines & Humphrey, 2022).

For numeric variables, predictive mean matching and Bayesian linear regression methods were used. For zero-inflated numeric variables that met count data criteria (integer and positive values), a zero-inflated Poisson method was used. For dichotomous variables, the logistic regression method was used. The chosen imputation methods were informed by previous simulation studies (Ge et al., 2023; Jadhav et al., 2019; Jia & Wu, 2022) and diagnostic plots, such as observed-by-imputed data distributions. The imputed data were limited to the minimum and maximum values of the respective scales.

Thirty imputations were conducted with 20 iterations each, meaning that 30 imputed datasets were calculated, the final values of which were decided after each dataset was estimated 20 times. The number of imputations was informed by the linear rule (White et al., 2011) which robustly estimates the minimum imputations required for datasets containing ≤30% missing data (Von Hippel, 2020). The analyses, described below, were conducted with each imputed dataset and the results were pooled via Rubin's Rules (Rubin, 1987). No auxiliary variables were included in the imputation due to insufficient correlations with imputed variables (r<0.3). Auxiliaries only reduce bias in imputed data when these correlate at least moderately (*r*≥0.4) with imputed variables (Collins et al., 2001), otherwise these may introduce additional bias (Hardt et al., 2012).

Convergence was observed for all variables as within-iteration variance of imputed data was greater than between-iteration variance (see Figure 10). Broadly similar distributions were also observed between the observed and imputed datasets (see Figure 11). Additionally, the descriptive statistics for the analysed variables were similar before and after the imputation (Tables 1 and 2), supporting

the credibility of the imputed data. On the basis of these diagnostic tests, the results of the imputation were considered robust.

References:

Bhaskaran, K., & Smeeth, L. (2014). What is the difference between missing completely at random and missing at random?. *International Journal of Epidemiology*, *43*(4), 1336–1339. https://doi.org/10.1093/ije/dyu080

Collins, L. M., Schafer, J. L., & Kam, C. M. (2001). A comparison of inclusive and restrictive strategies in modern missing data procedures. *Psychological Methods*, *6*(4), 330. https://doi.org/10.1037/1082-989x.6.4.330

Ge, Y., Li, Z., & Zhang, J. (2023). A simulation study on missing data imputation for dichotomous variables using statistical and machine learning methods. *Scientific Reports*, *13*(1), 9432. https://doi.org/10.1038/s41598-023-36509-2

Hardt, J., Herke, M., & Leonhart, R. (2012). Auxiliary variables in multiple imputation in regression with missing X: a warning against including too many in small sample research. *BMC Medical Research Methodology*, *12*(1), 184. https://doi.org/10.1186/1471-2288-12-184

Jadhav, A., Pramod, D., & Ramanathan, K. (2019). Comparison of performance of data imputation methods for numeric dataset. *Applied Artificial Intelligence*, *33*(10), 913-933. https://doi.org/10.1080/08839514.2019.1637138

Jia, F., & Wu, W. (2023). A comparison of multiple imputation strategies to deal with missing nonnormal data in structural equation modeling. *Behavior Research Methods*, *55*(6), 3100-3119. https://doi.org/10.3758/s13428-022-01936-y

Kleinke, K., & Reinecke, J. (2019). Countimp version 2 – A multiple imputation package for incomplete count data [Technical Report]. University of Siegen, Department of Education Studies; Psychology. https://kkleinke.de/countimp

Lines, L.. & Humphrey, J. (2022, January 1). *Imputing Race & Ethnicity: Part 1.* The Medical Care Blog. https://www.themedicalcareblog.com/imputing-race-ethnicity-1/

Madley-Dowd, P., Hughes, R., Tilling, K., & Heron, J. (2019). The proportion of missing data should not be used to guide decisions on multiple imputation. *Journal of Clinical Epidemiology*, *110*, 63–73. https://doi.org/10.1016/j.jclinepi.2019.02.016

Robitzsch, A. & Grund, S. (2024). miceadds: Some Additional Multiple Imputation Functions. R package version 3.17-44. https://CRAN.R-project.org/package=miceadds

Rubin, D. B. (1987). Multiple imputation for nonresponse in surveys. John Wiley & Sons. onlinelibrary.wiley.com

Silva, G. C., Trivedi, A. N., & Gutman, R. (2019). Developing and evaluating methods to impute race/ethnicity in an incomplete dataset. *Health Services and Outcomes Research Methodology*, *19*(2), 175-195. https://doi.org/10.1007/s10742-019-00200-9

Tierney, N., & Cook, D. (2023). Expanding tidy data principles to facilitate missing data exploration, visualization and assessment of imputations. *Journal of Statistical Software*, *105*, 1-31. https://doi.org/10.18637/jss.v105.i07

Van Buuren, S., & Groothuis-Oudshoorn, K. (2011). mice: Multivariate imputation by chained equations in R. *Journal of Statistical Software*, *45*, 1-67. https://doi.org/10.18637/jss.v045.i03

Von Hippel, P. T. (2020). How many imputations do you need? A two-stage calculation using a quadratic rule. *Sociological Methods & Research*, *49*(3), 699-718. https://doi.org/10.1177/0049124117747303

White, I. R., Royston, P., & Wood, A. M. (2011). Multiple imputation using chained equations: issues and guidance for practice. *Statistics in Medicine*, *30*(4), 377-399. https://doi.org/10.1002/sim.4067

Wickham, H. (2016). Data Analysis. In: ggplot2. Use R!. Springer, Cham. https://doi.org/10.1007/978-3-319-24277-4_9

Woods, A. D., Gerasimova, D., Van Dusen, B., Nissen, J., Bainter, S., Uzdavines, A., ... & Elsherif, M. M. (2024). Best practices for addressing missing data through multiple imputation. *Infant and Child Development*, *33*(1), e2407. https://doi.org/10.1002/icd.2407

Table 1. Frequency of categorical variable levels before and after multiple imputation

Variable	Before imputation N (%)	After imputation N (%)
Socioeconomic status		
Below national average	63 (18.50)	65 (18.89)
Around national average	63 (18.50)	65 (18.93)
Above national average	186 (54.70)	189 (55.93)
Prefer not to say	21 (6.20)	21 (6.25)
Missing	7 (2.10)	0 (0)
Verbal ability		
< 30 words/signs	121 (35.60)	122 (35.13)
> 30 words/signs	215 (63.20)	218 (64.87)
Missing	4 (1.20)	0 (0)

Table 2. Descriptive statistics for continuous variables before and after the multiple imputation

Variable	Before imputation[a] Mean (*SD*)	Range	After imputation Mean (*SD*)	Range
CSHQ bedtime resistance	8.61 (*2.89*)	6 – 18	8.62 (*2.81*)	6 – 18
CSHQ sleep onset delay	1.60 (*0.76*)	1 – 3	1.57 (*0.74*)	1 – 3
CSHQ sleep duration	4.61 (*1.80*)	3 – 9	4.60 (*1.79*)	3 – 9
CSHQ sleep anxiety	5.96 (*2.04*)	4 – 12	5.97 (*2.04*)	4 – 12
CSHQ night awakenings	5.03 (*1.77*)	3 – 9	5.04 (*1.77*)	3 – 9
CSHQ parasomnias	10.22 (*2.38*)	7 – 19	10.24 (*2.40*)	7 – 19
CSHQ sleep-disordered breathing	3.92 (*1.39*)	3 – 9	3.95 (*1.42*)	3 – 9
CSHQ daytime sleepiness	11.95 (3.00)	8 – 24	11.94 (*2.98*)	8 – 24
TAQ overactivity	17.16 (*9.65*)	0 – 36	17.16 (*9.62*)	0 – 36
TAQ impulsivity	16.02 (*7.01*)	0 – 24	15.89 (*6.96*)	0 – 24
CBQ self-injury	2.93 (3.63)	0 – 12	2.84 (*3.58*)	0 – 12
CBQ aggression	3.81 (*3.93*)	0 – 14	3.79 (*3.92*)	0 – 14
CBQ destruction of property	3.13 (*3.82*)	0 – 13	3.02 (*3.78*)	0 – 13
MIPQ total	36.50 (*6.58*)	10 – 48	36.46 (*6.57*)	10 – 48
ClASP-ID anxiety	37.12 (*16.61*)	14 – 96	36.92 (*16.65*)	14 – 96
M-CHAT-R + SCQ-C *z*-scores[b]	0.65 (*0.99*)	-1.52 – 3.04	0.64 (0.99)	-1.52 – 3.04
HSSS total	4.14 (*10.14*)	0 – 45	4.18 (*10.14*)	0 – 45
HQ month total	4.32 (*3.96*)	0 – 21	4.49 (*3.90*)	0 – 21

Note. CSHQ: Children's Sleep Habits Questionnaire. TAQ: The Activity Questionnaire. CBQ: Challenging Behaviour Questionnaire. MIPQ: Mood Interest and Pleasure Questionnaire. ClASP-ID: Clinical Anxiety Screen for people with Severe to Profound Intellectual Disability. M-CHAT-R: Modified Checklist for Autism in Toddlers – Revised. SCQ: Social Communication Questionnaire. HSSS: Hague Seizure Severity Scale. HQ: Health Questionnaire.

[a] Descriptive statistics presented following pairwise deletion. The proportions of missing data for each variable are presented in Appendix 4.7.

[b] Since the M-CHAT-R and SCQ-C were completed by different age groups, the total scores from both measures were converted to *z*-scores and combined into one variable representing autism-related characteristics.

Figure 1: Rates of missingness for analysed variables

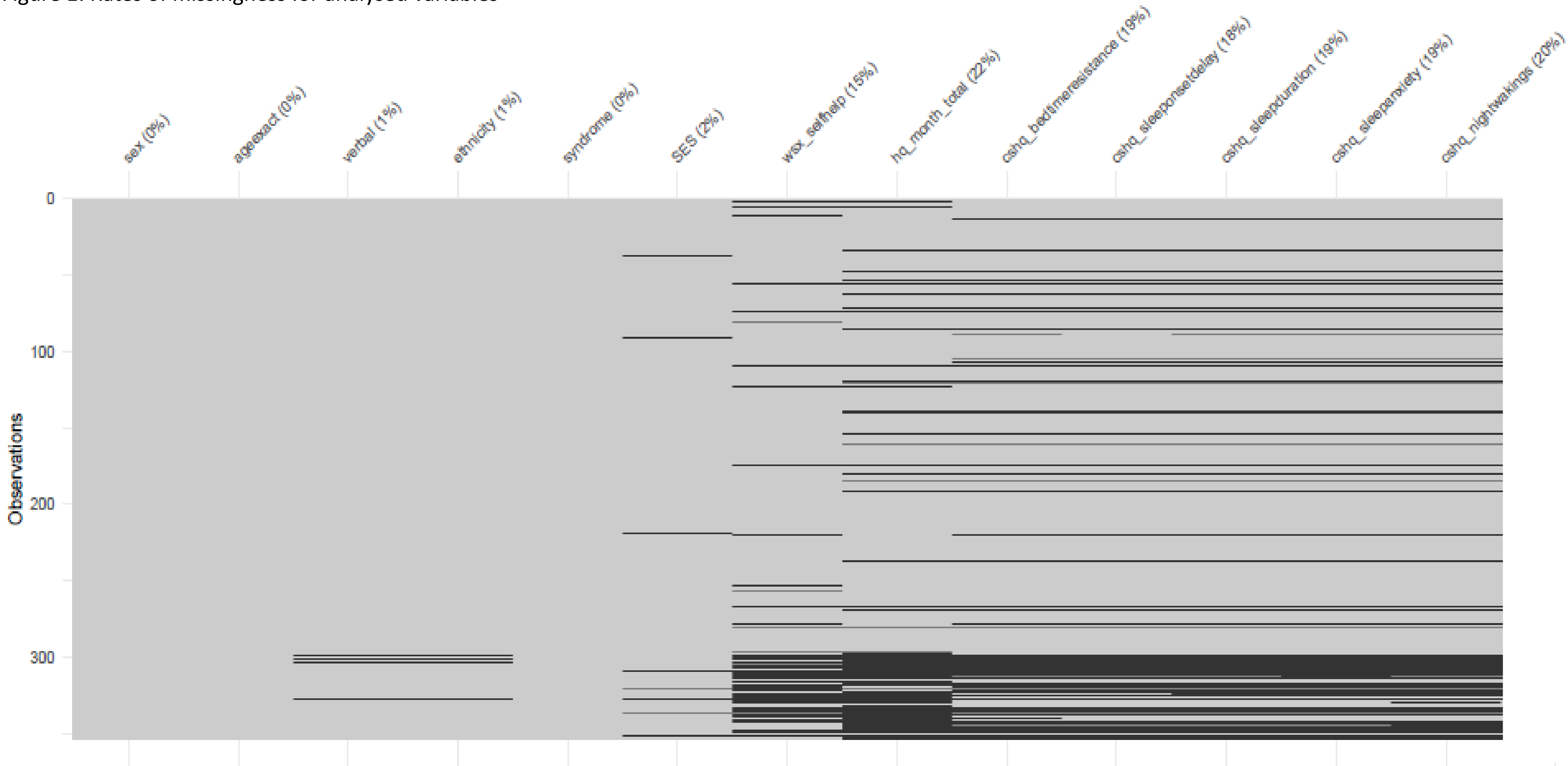

Note. Proportion of missing data per variable presented alongside variable labels.

Figure 1 (continued): Rates of missingness for analysed variables

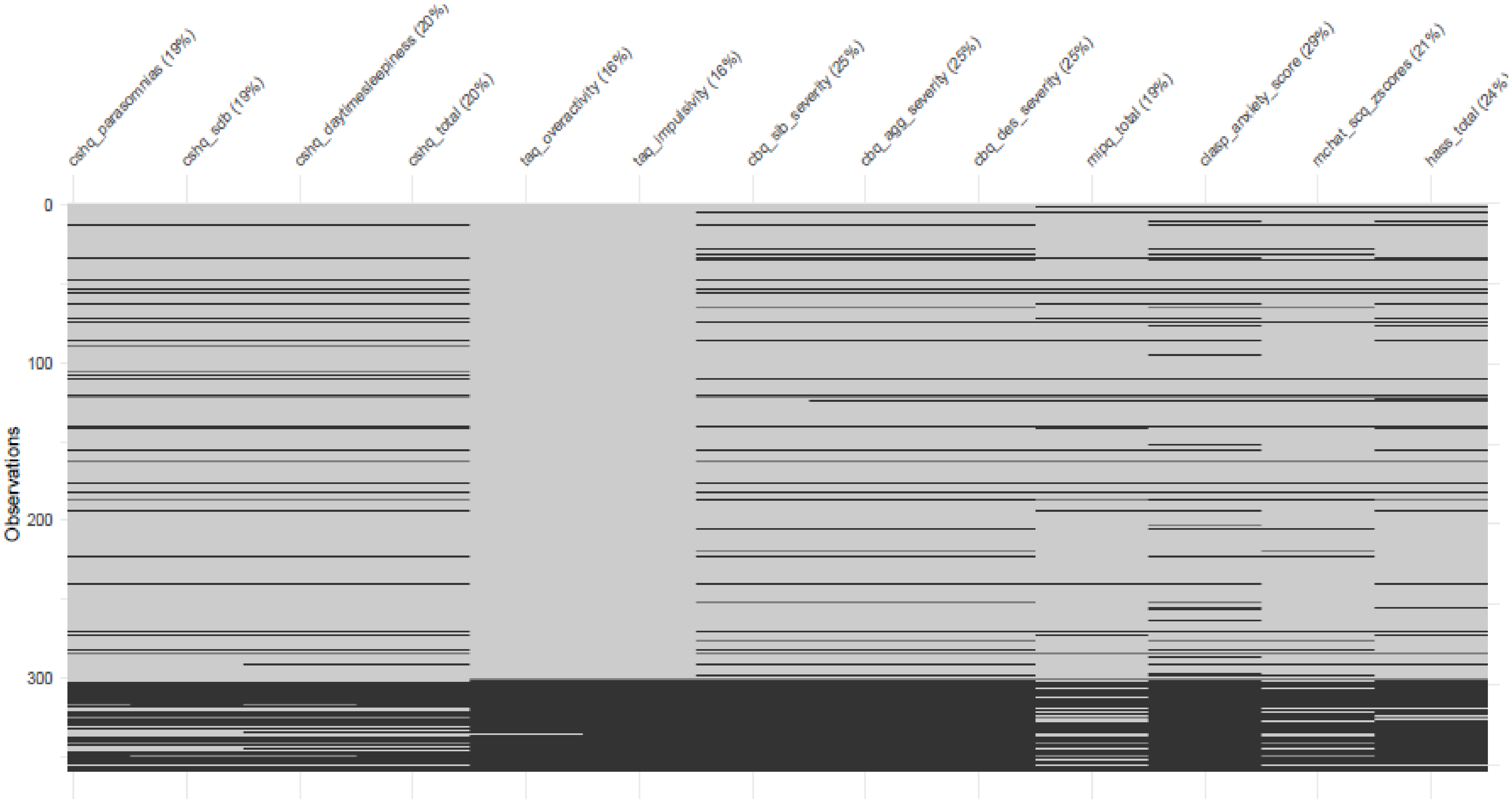

Note. Proportion of missing data per variable presented alongside variable labels.

Figure 2: Distributions of observed and missing data for the analysed variables, according to children's age.

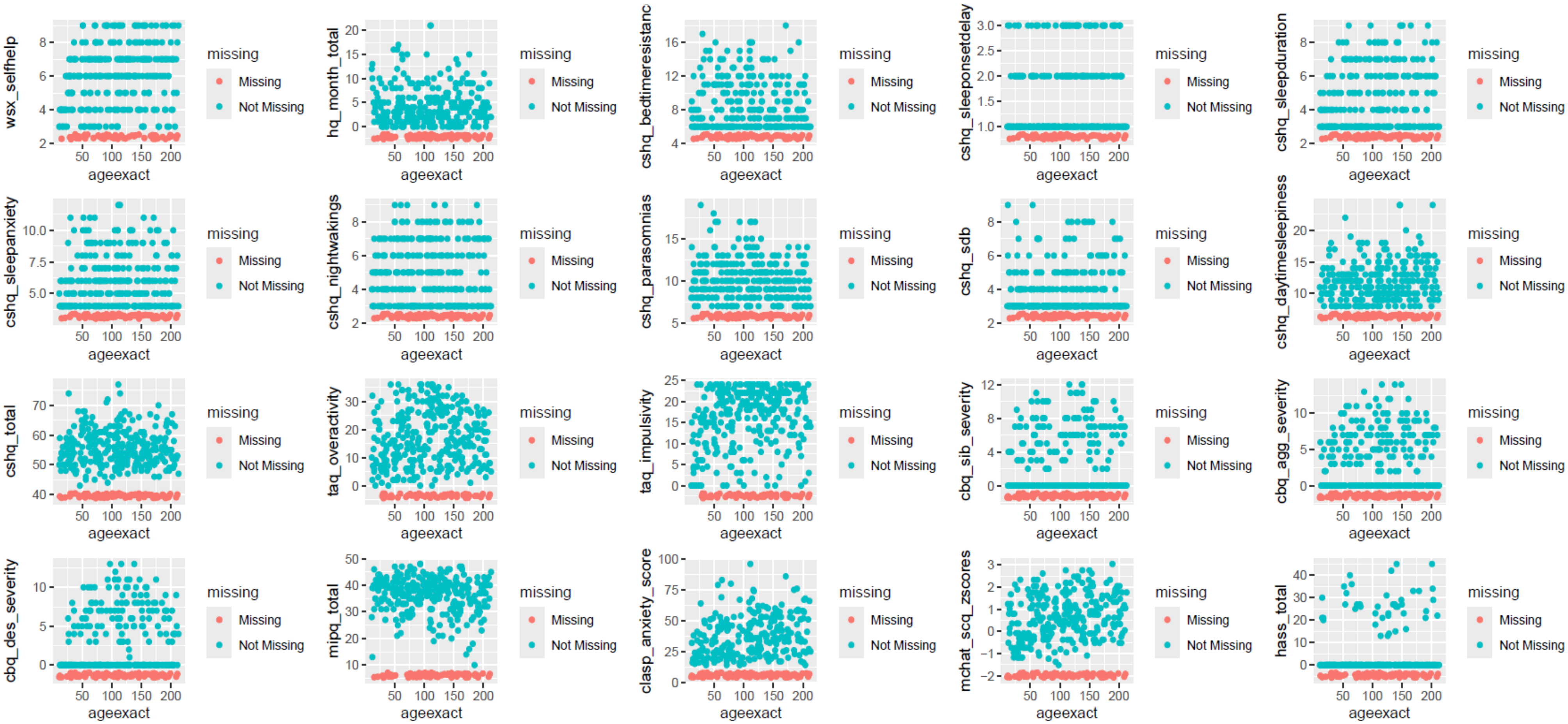

Note. Green points represent individuals with observed data for both variables. Red points along the x-axis represent individuals with observed data for the x-axis variable, but not the y-axis variable.

Figure 3: Proportion of missing data for each analysed variable, according to level of adaptive functioning.

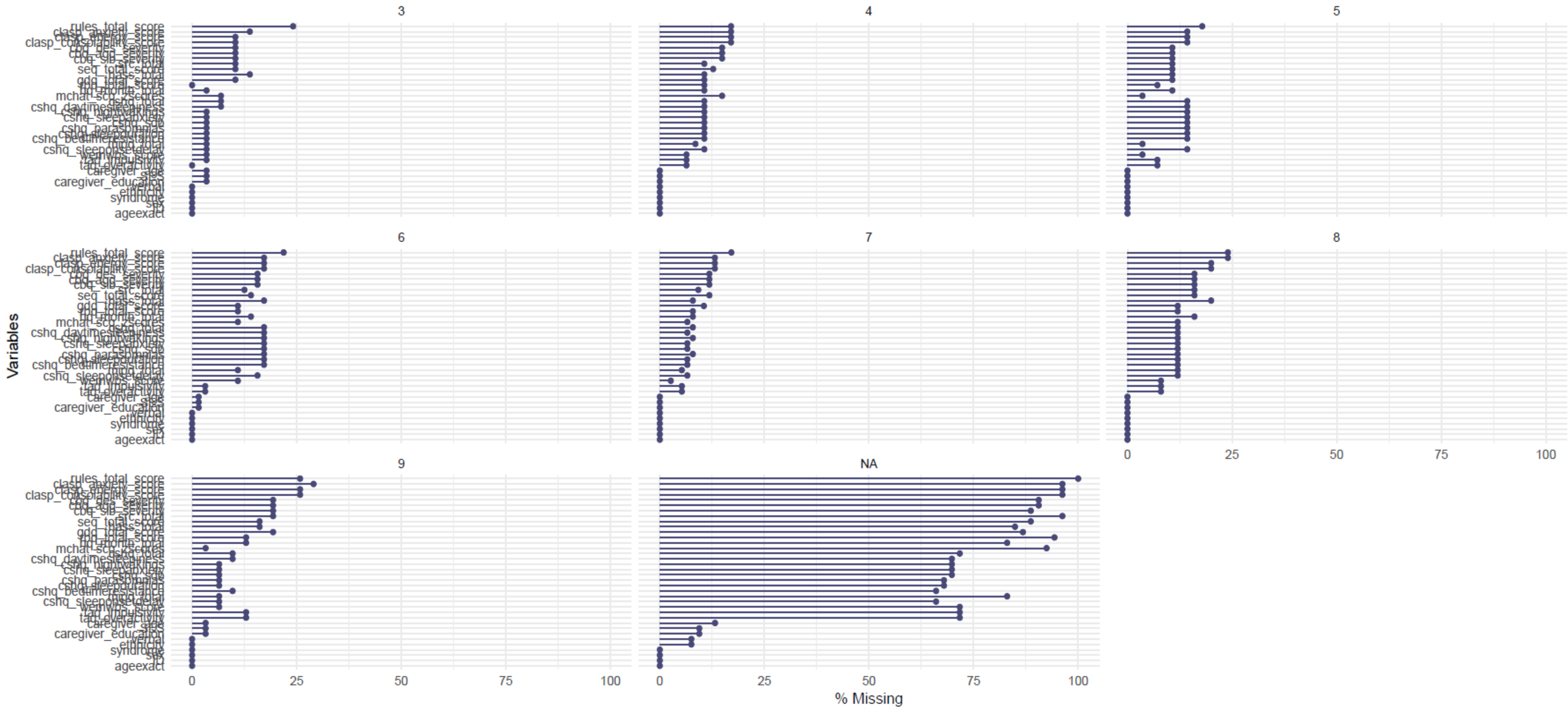

Note. Higher score reflects higher day-to-day functioning.

Figure 4: Proportion of missing data for each analysed variable, according to syndrome diagnosis.

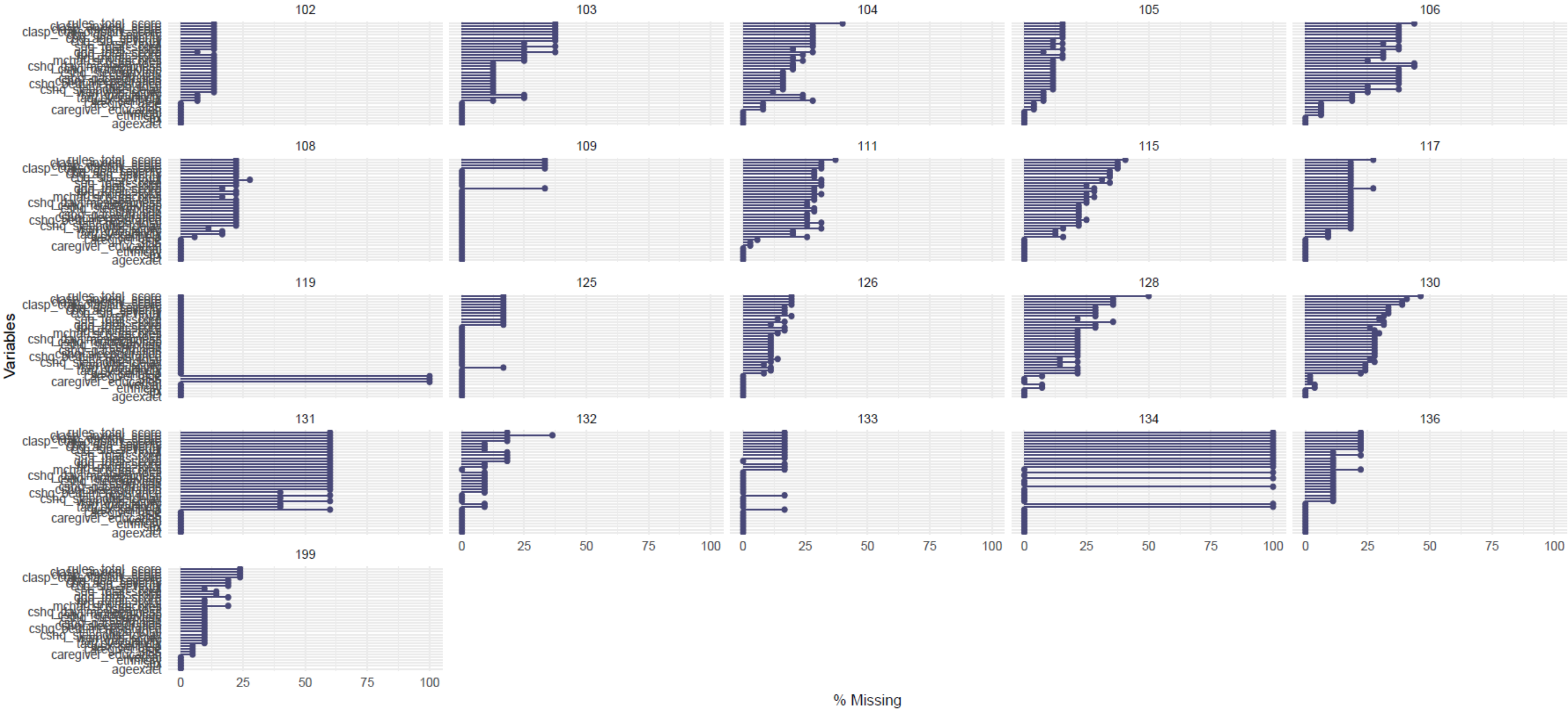

Note. 102: Angelman syndrome. 103: Cri du Chat syndrome. 104: Cornelia de Lange syndrome. 105: Fragile X syndrome. 106: Prader-Willi syndrome. 108: Smith-Magenis syndrome. 109: Down syndrome. 111: Rubinstein-Taybi syndrome. 115: Kleefstra syndrome. 117: tuberous sclerosis complex. 119: 19p13. 125: Pallister-Killian syndrome. 126: SATB2-associated syndrome. 128: Coffin-Siris syndrome. 130: KBG syndrome. 131: SYNGAP1 syndrome. 132: Wiedemann-Steiner syndrome. 133: Wolf-Hirschhorn syndrome. 134: 15q. 136: Jansen de Vries syndrome. 199: 'Other' syndrome not listed.

Figure 5: Proportion of missing data for each analysed variable, according to children's ethnicity.

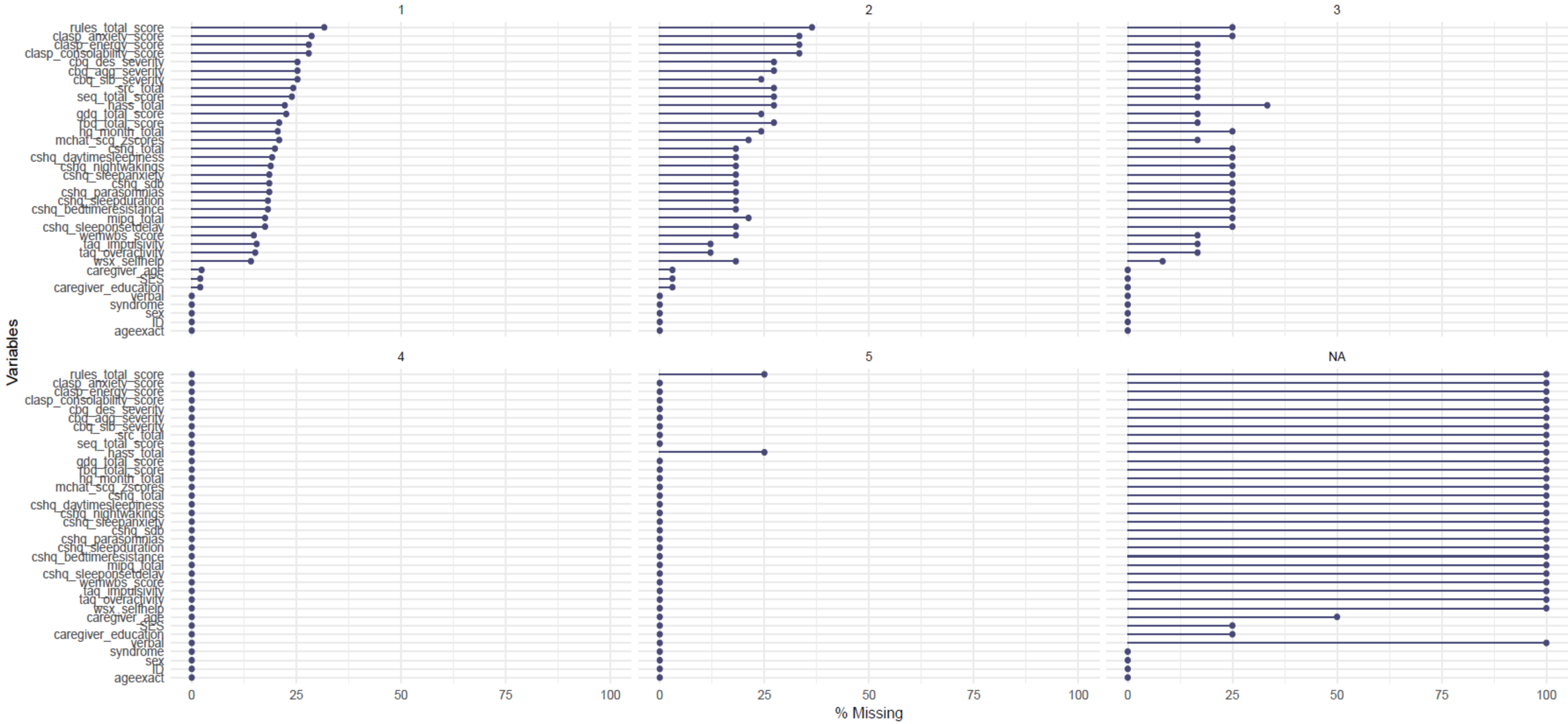

Note. 1: White. 2: Mixed ethnicity. 3: Asian or Asian British. 4: Black, African, Caribbean, or Black British. 5: 'Other' ethnicity not listed.

Figure 6: Proportion of missing data for each analysed variable, according to socioeconomic status.

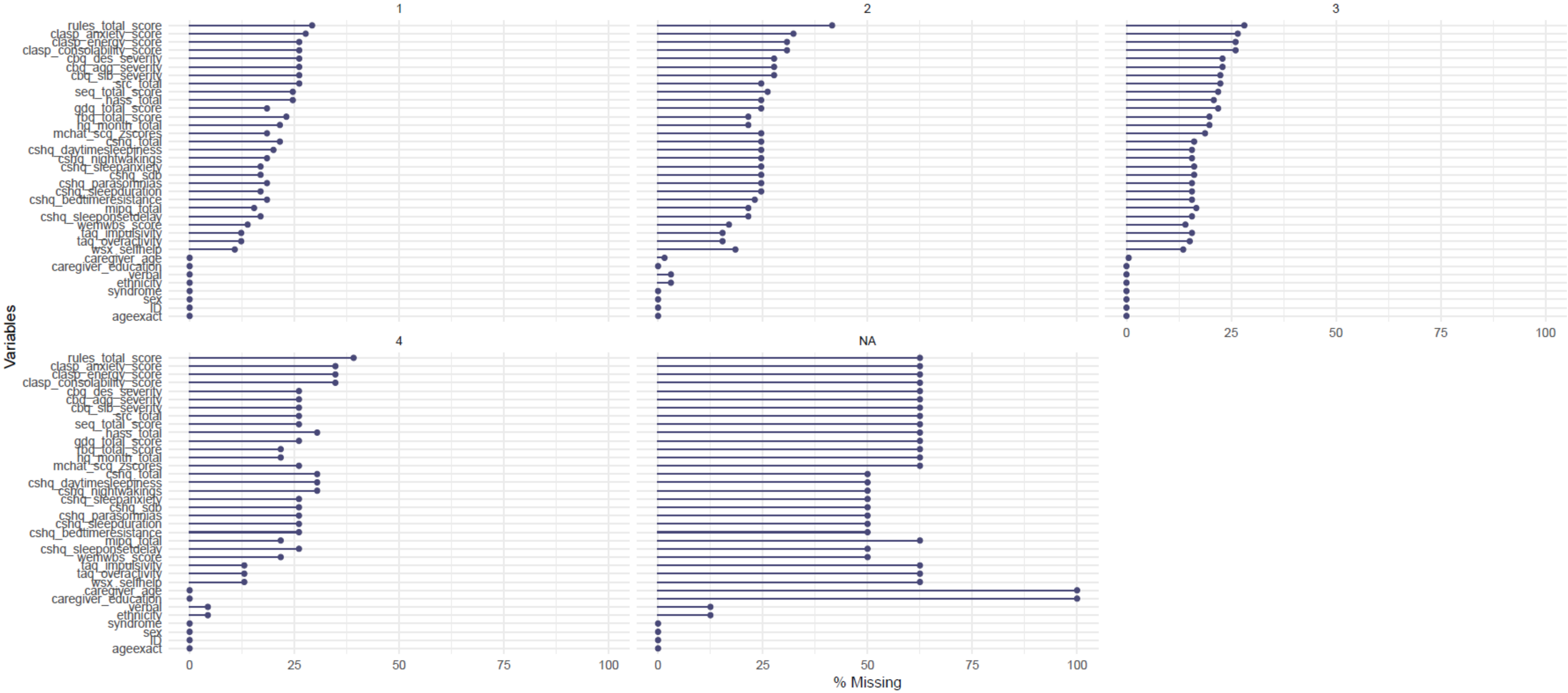

Note. 1: Below national average. 2: Similar to national average. 3: Above the national average. 4: Prefer not to say.

Figure 7: Distributions of observed and missing data for the analysed variables, according to caregivers' age.

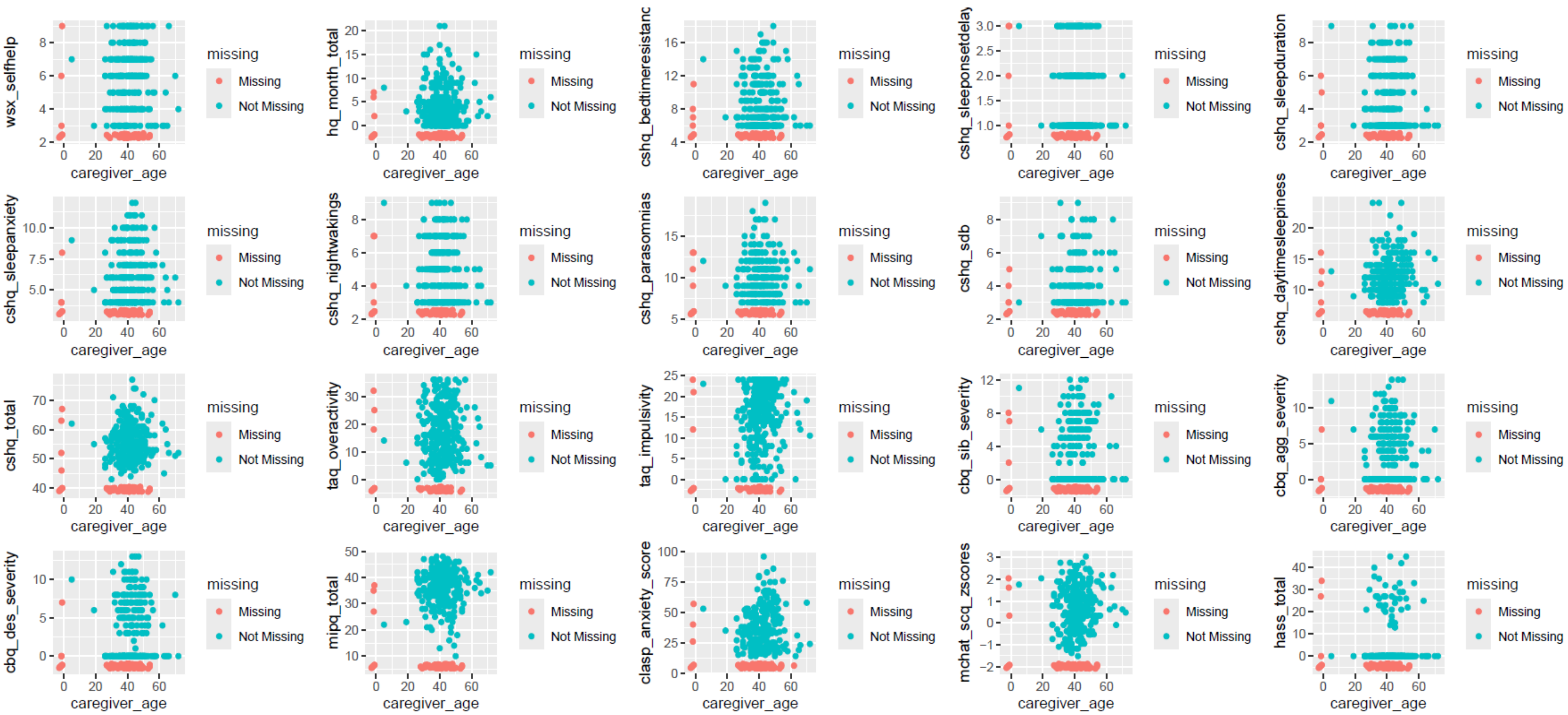

Note. Green points represent individuals with observed data for both variables. Red points along the x-axis represent individuals with observed data for the x-axis variable, but not the y-axis variable. Red points along the y-axis represent individuals with observed data for the y-axis variable, but not the x-axis variable.

Figure 8: Proportion of missing data for each analysed variable, according to caregiver education.

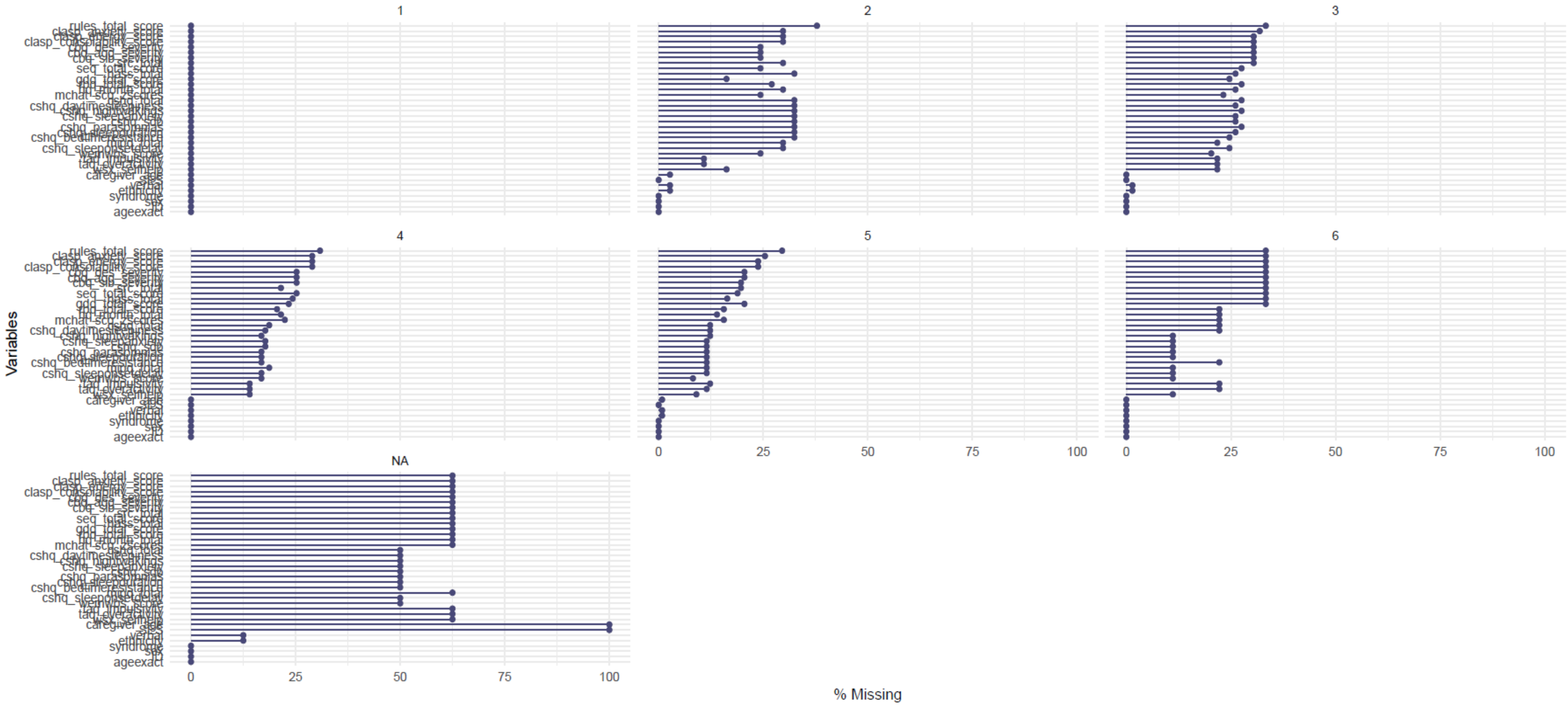

Note. 1: No formal education. 2: Secondary school, GCSEs, or equivalent. 3: College, sixth form, A-levels, or equivalent. 4: University, undergraduate degree, or equivalent. 5: University, postgraduate, or equivalent. 6: 'Other' qualification not listed.

Figure 9: Proportion of missing data for each analysed variable, according to country of residence.

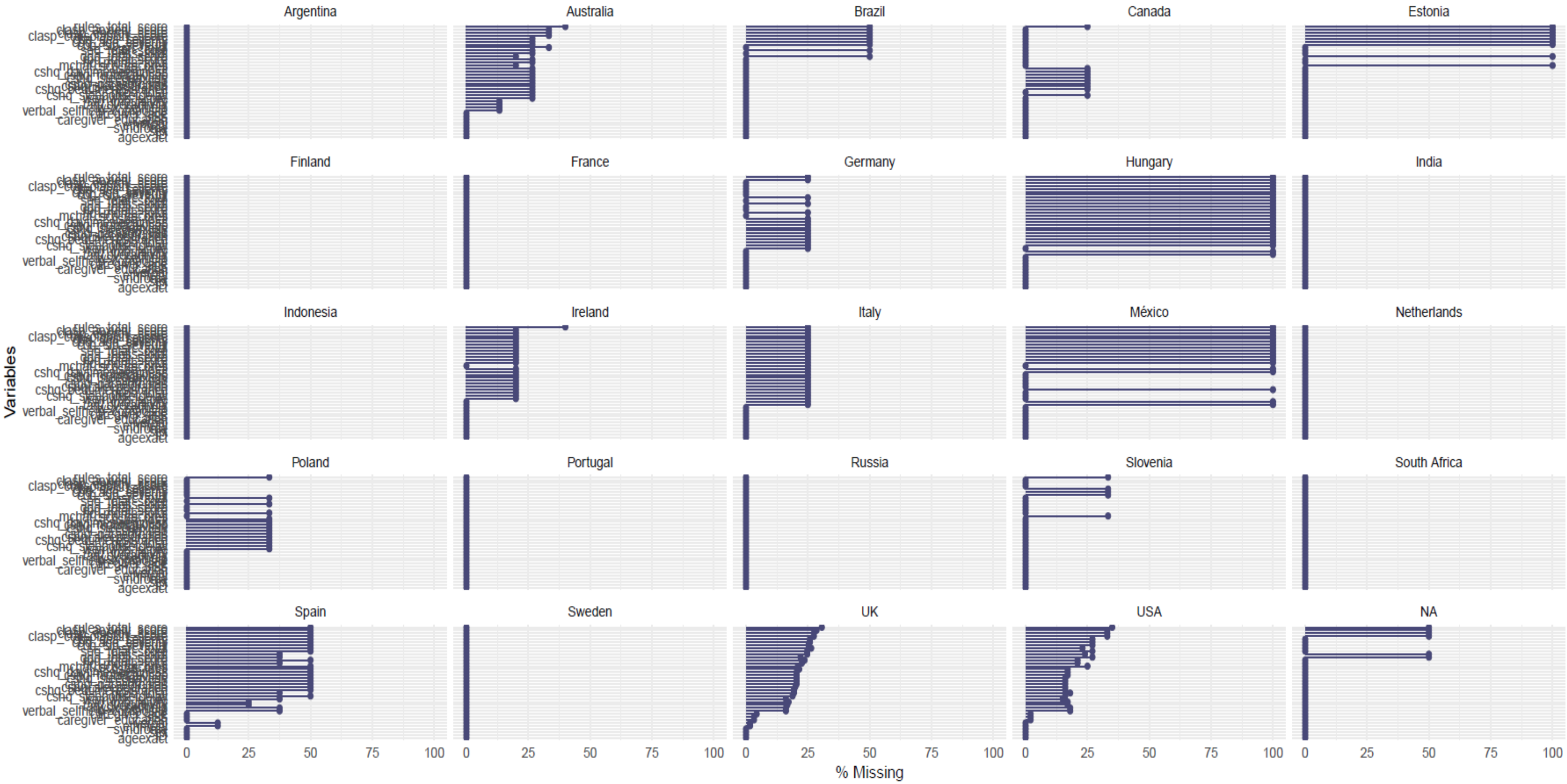

Note. UK: United Kingdom. USA: United States of America.

Figure 10: Means and standard deviations for the analysed variables, within each imputed dataset across the 20 iterations.

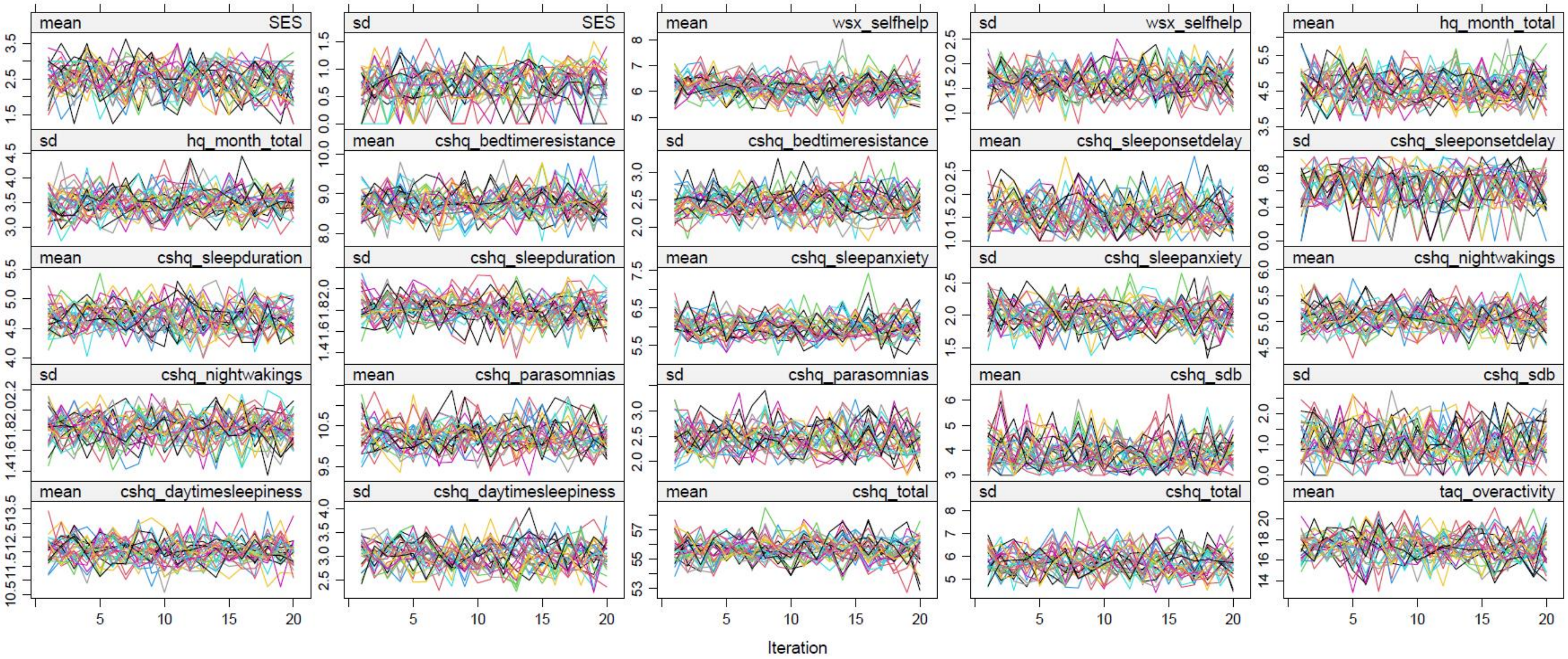

Note. Each coloured line represents an imputed dataset (30 imputed datasets in total, 30 lines). Each imputed dataset is estimated 20 times (once per iteration, 20 iterations) before the final values for each imputed dataset are chosen.

Figure 11 (continued): Means and standard deviations for the analysed variables, within each imputed dataset across the 20 iterations.

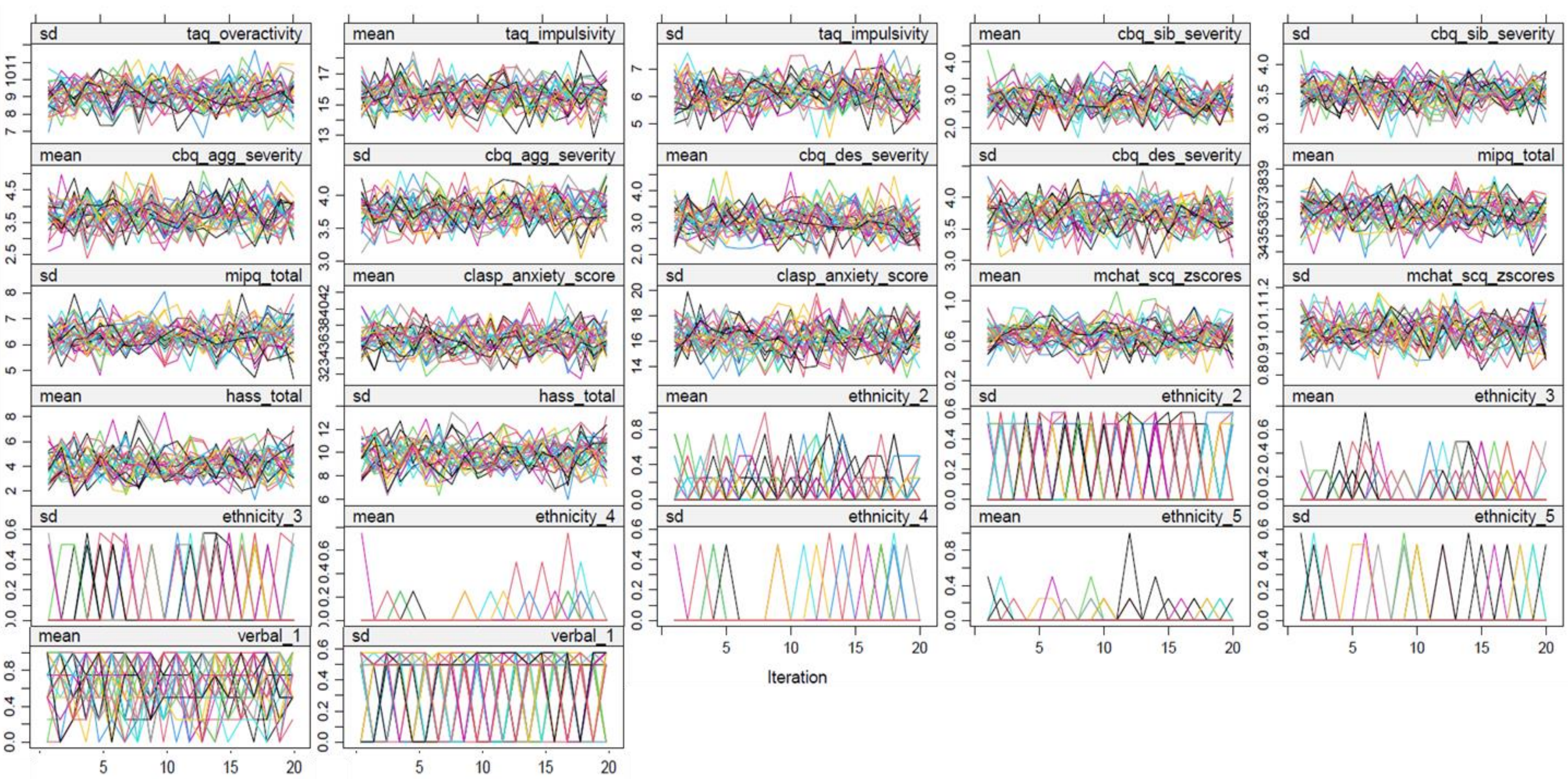

Note. Each coloured line represents an imputed dataset (30 imputed datasets in total, 30 lines). Each imputed dataset is estimated 20 times (once per iteration, 20 iterations) before the final values for each imputed dataset are chosen.

Figure 12: Distributions of observed and imputed datasets, separated by each analysed variable.

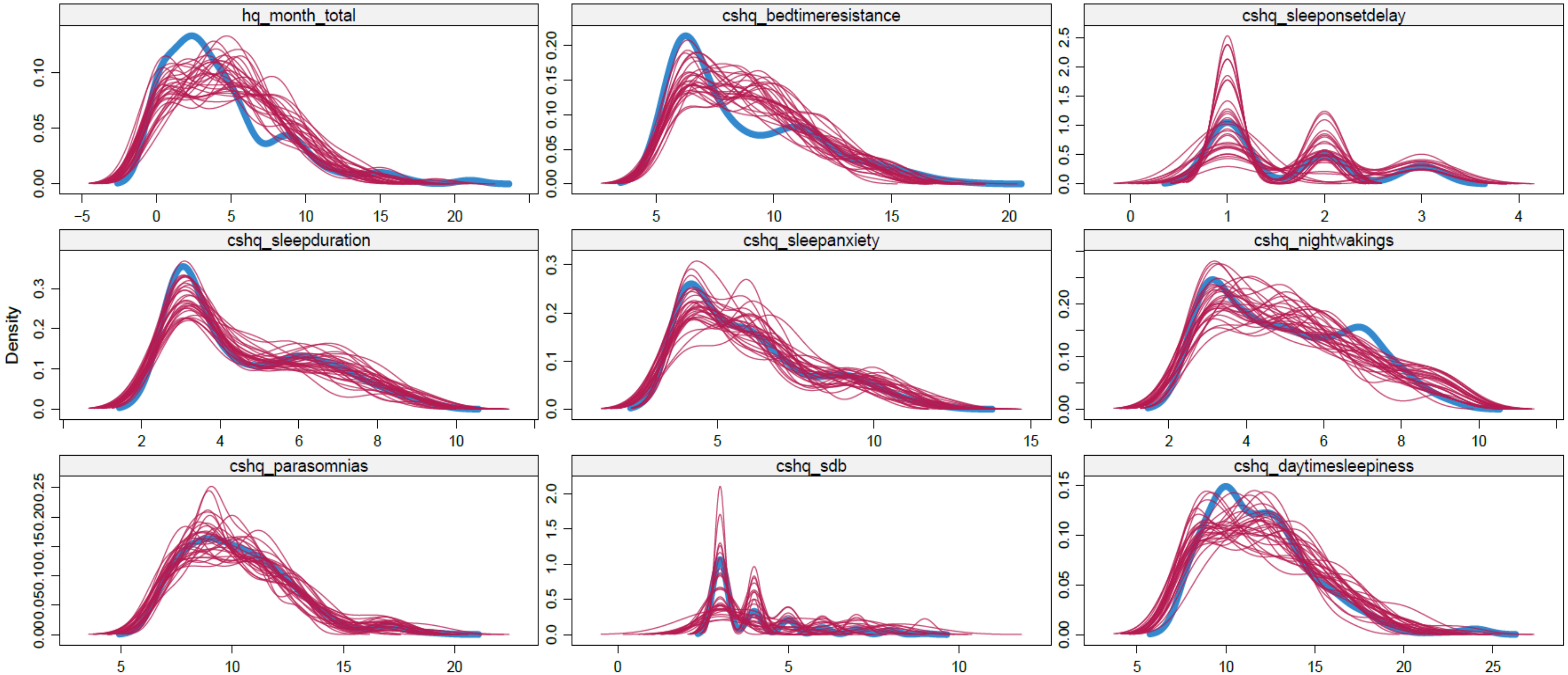

Note. Blue line represents observed dataset. Each red line represents one of the 30 imputed datasets. The x-axis represents scores for each analysed variable.

Figure 12 (continued): Distributions of observed and imputed datasets, separated by each analysed variable.

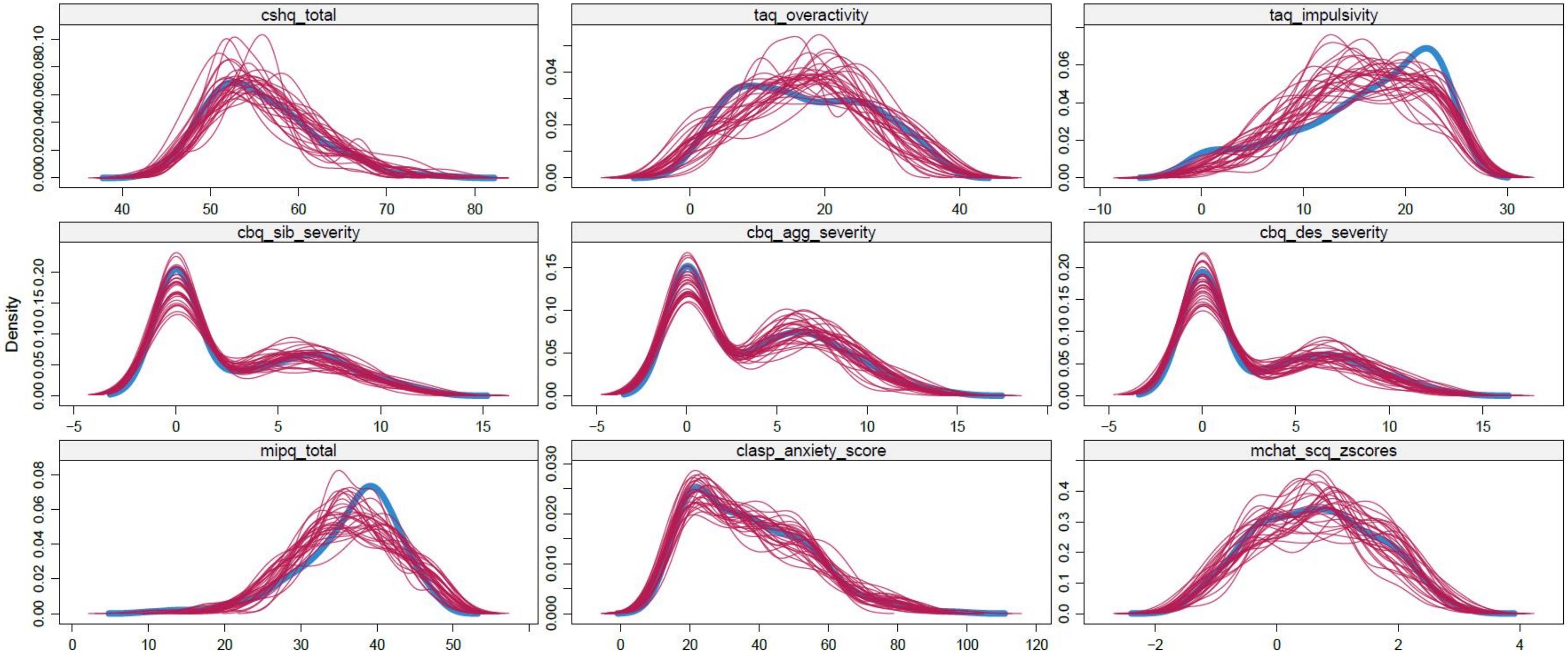

Note. Blue line represents observed dataset. Each red line represents one of the 30 imputed datasets. The x-axis represents scores for each analysed variable.

Figure 12 (continued): Distributions of observed and imputed datasets, separated by each analysed variable.

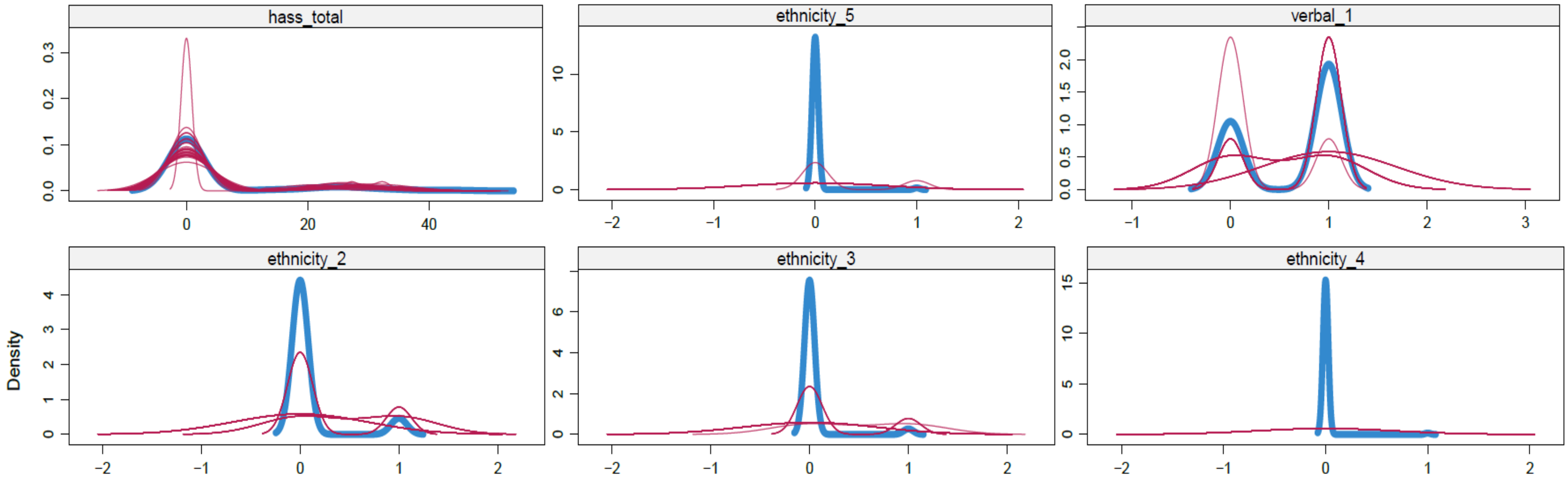

Note. Blue line represents observed dataset. Each red line represents one of the 30 imputed datasets. The x-axis represents scores for each analysed variable.
