# Supplementary material for: The interactions between sleep difficulties and clinical features in children with rare genetic syndromes: a structural equation modelling approach
Source: BMC Pediatr. 2026 Jun 5;26:740. doi: 10.1186/s12887-026-07099-5 (PMC13471272; doi:10.1186/s12887-026-07099-5)
Supplement: Supplementary file 6 [file 12887_2026_7099_MOESM6_ESM.pdf]

Additional file 6: Results following multiple imputation

Table 1. Demographic characteristics of the final sample, after multiple imputation, separated by genetic syndrome.

Syndrome *(N)*	Age in years M*(SD)*	Male/ female (N)	Ethnicity[a] Wh (N)	Mi (N)	Asi (N)	Bl (N)	Oth (N)	Socioeconomic status[a] <NA (N)	~NA (N)	>NA (N)	ND (N)
AS *(14)*	13.76 *(3.70)*	5/9	11	3	0	0	0	4	1	9	0
CdC *(8)*	6.29 *(3.66)*	3/5	7	0	1	0	0	0	2	6	0
CdLS *(23)*	9.73 *(4.37)*	10/13	19	1	2	1	0	4	6	10	3
CSS *(14)*	6.79 *(3.93)*	6/8	6	5	1	0	2	3	7	4	0
DS *(3)*	5.31 *(5.06)*	3/0	3	0	0	0	0	0	1	2	0
FXS *(23)*	11.38 *(4.08)*	20/3	18	1	2	1	1	3	0	19	1
JdVS *(9)*	8.50 *(2.72)*	5/4	9	0	0	0	0	1	1	5	2
KBGS *(53)*	7.74 *(3.76)*	33/20	46	3	2	0	0	10	16	24	3
KS *(32)*	9.35 *(5.18)*	16/16	29	3	0	0	0	8	4	16	4
PKS *(6)*	6.33 *(4.24)*	3/3	6	0	0	0	0	2	1	2	1
PWS *(16)*	7.09 *(4.48)*	9/7	12	2	1	0	1	6	0	9	1
RTS *(33)*	9.58 *(5.47)*	15/18	30	3	0	0	0	6	5	22	0
SAS *(36)*	9.33 *(3.85)*	23/13	28	5	3	0	0	6	6	21	3
SMS *(17)*	10.54 *(4.64)*	13/4	17	0	0	0	0	3	4	9	1
SYNGAP1 *(4)*	13.79 *(1.01)*	2/2	3	0	0	1	0	0	1	3	0
TSC *(11)*	8.42 *(4.58)*	5/6	9	2	0	0	0	2	1	7	1
WHS *(6)*	7.17 *(2.18)*	2/4	6	0	0	0	0	0	1	4	1
WSS *(10)*	10.33 *(3.38)*	6/4	9	0	1	0	0	3	1	6	0
Other *(22)*[b]	7.81 *(4.44)*	11/11	20	2	0	0	0	4	4	13	1

Table 1 (Continued). Demographic characteristics of the final sample, after multiple imputation, separated by genetic syndrome.

Syndrome *(N)*	Level of intellectual disability					
	None (N)	Mild (N)	Mod (N)	Sev (N)	Prof (N)	Unk (N)
AS *(14)*	1	0	0	5	3	5
CdC *(8)*	0	2	1	2	1	2
CdLS *(23)*	0	3	7	10	1	2
CSS *(14)*	2	2	5	3	0	2
DS *(3)*	0	0	0	1	0	2
FXS *(23)*	3	0	9	7	1	3
JdVS *(9)*	0	1	4	3	0	1
KBGS (*53)*	13	10	21	2	0	7
KS *(32)*	3	3	10	8	5	3
PKS *(6)*	0	0	0	1	4	1
PWS *(16)*	5	1	5	2	0	2
RTS *(33)*	2	2	9	12	1	7
SAS *(36)*	2	4	10	9	6	5
SMS *(17)*	0	1	5	5	0	3
SYNGAP1 *(4)*	0	0	0	1	3	0
TSC *(11)*	3	2	2	4	0	0
WHS *(6)*	0	0	0	3	2	1
WSS *(10)*	0	4	4	0	1	1
Other *(22)*[b]	6	2	6	3	1	4

Note. M: mean. Wh: White. Mi: Mixed ethnicity or multiple ethnic groups. Asi: Asian or Asian British. Bl: Black, African, Caribbean, or Black British. Oth: Other. NA: national average household income. ND: Not disclosed. AS: Angelman syndrome. CdC: Cri-du-Chat syndrome. CdLS: Cornelia de Lange syndrome. CSS: Coffin-Siris syndrome. DS: Down syndrome. FXS: Fragile X syndrome. JdVS: Jansen-de Vries syndrome. KBGS: KBG syndrome. KS: Kleefstra syndrome. PKS: Pallister-Killian syndrome. PWS: Prader-Willi syndrome. RTS: Rubinstein-Taybi syndrome. SAS: SATB2-associated syndrome. SMS: Smith-Magenis syndrome. TSC: tuberous sclerosis complex. WHS: Wolf-Hirschhorn syndrome. WSS: Wiedemann-Steiner syndrome. Mod: Moderate. Sev: Severe. Prof: Profound. Unk: Unknown.

[a] Imputed data for ethnicity (missingness: 1%) and socioeconomic status (missingness: 2%) are presented.

[b] The 'Other' group includes 19 distinct syndromes. Details of these syndromes and syndrome-specific demographics are in Table 2.

Table 2. Demographic characteristics of the children with ‘other’ rare genetic syndromes, after multiple imputation.

Syndrome *(N)*	Age (Y)	Male/ female (N)	Ethnicity					Socioeconomic status			
			Wh (N)	Mi (N)	As (N)	Bl (N)	Oth (N)	<NA (N)	~NA (N)	>NA (N)	ND (N)
19p13.13 deletion *(1)*	16.93	0/1	1	0	0	0	0	0	0	0	1
15q *(1)*	2.50	0/1	1	0	0	0	0	1	0	0	0
10q22-10q23 del *(1)*	4.67	1/0	1	0	0	0	0	0	1	0	0
15q11.2q13.1 & 17p13.3 mdup *(1)*	2.33	1/0	1	0	0	0	0	0	0	1	0
16p11.2 dup *(2)*	5.25, 9.5	0/2	2	0	0	0	0	1	0	1	0
16p12.12 mdel *(1)*	6.17	0/1	1	0	0	0	0	0	0	1	0
1q21.1q21.2 *(2)*	13.00, 9.92	0/2	1	1	0	0	0	2	0	0	0
1q42 dup *(1)*	5.42	0/1	1	0	0	0	0	0	0	1	0
22q11.2 del *(1)*	7.92	1/0	1	0	0	0	0	0	0	1	0
2p16.3 mdel *(1)*	5.08	1/0	1	0	0	0	0	0	0	1	0
8p inv dup/del *(1)*	17.25	1/0	1	0	0	0	0	0	0	1	0
CACNA1A *(1)*	6.17	0/1	1	0	0	0	0	0	0	1	0
GS *(1)*	12.17	1/0	1	0	0	0	0	0	1	0	0
JS *(1)*	7.75	1/0	1	0	0	0	0	0	0	1	0
Med13L *(1)*	14.33	0/1	1	0	0	0	0	0	0	1	0
RSS *(2)*	5.25, 1.08	0/2	2	0	0	0	0	0	1	1	0
Shank 2 del *(1)*	9.83	1/0	1	0	0	0	0	0	0	1	0
SWAN *(2)*	2.25 & 14.67	2/0	1	1	0	0	0	0	0	1	1
WS *(1)*	9.25	1/0	1	0	0	0	0	0	1	0	0

Note. As: Asian or Asian British. Bl: Black, African, Caribbean, or Black British. ID: intellectual disability. del: deletion. dup: duplication. GS: Glass syndrome. inv: inverted. JS: Jacobs syndrome. mdel: microdeletion. mdup: microduplication. Mi: Mixed ethnicity or multiple ethnic groups. Mild: Mild intellectual disability. Mod: Moderate intellectual disability. NA: national average household income. ND: Not disclosed. Oth: Other. Prof: Profound intellectual disability. RSS: Russell Silver syndrome. SES: socioeconomic status. Sev: Severe intellectual disability. SWAN: syndrome without a name. Unk: Unknown level of intellectual disability. Wh: White. WS: Weaver syndrome. Y: years.

Table 2 (Continued). Demographic characteristics of the children with 'other' rare genetic syndromes, after multiple imputation.

Syndrome *(N)*	Level of intellectual disability					
	None (N)	Mild (N)	Mod (N)	Sev (N)	Prof (N)	Unk (N)
19p13.13 deletion *(1)*	0	0	0	1	0	0
15q *(1)*	0	0	1	0	0	0
10q22-10q23 del *(1)*	0	0	0	1	0	0
15q11.2q13.1 & 17p13.3 mdup *(1)*	1	0	0	0	0	0
16p11.2 dup *(2)*	1	0	0	0	0	1
16p12.12 mdel *(1)*	0	0	0	0	0	1
1q21.1q21.2 *(2)*	0	1	1	0	0	0
1q42 dup *(1)*	0	0	1	0	0	0
22q11.2 del *(1)*	1	0	0	0	0	0
2p16.3 mdel *(1)*	0	0	0	0	0	1
8p inv dup/del *(1)*	0	0	0	0	1	0
CACNA1A *(1)*	0	0	1	0	0	0
GS *(1)*	0	0	0	0	0	1
JS *(1)*	0	0	1	0	0	0
Med13L *(1)*	0	0	0	1	0	0
RSS *(2)*	2	0	0	0	0	0
Shank 2 del *(1)*	0	0	1	0	0	0
SWAN *(2)*	1	0	0	1	0	0
WS *(1)*	0	1	0	0	0	0

Note. Mild: Mild intellectual disability. Mod: Moderate intellectual disability. Sev: Severe intellectual disability. Prof: Profound intellectual disability. Unk: Unknown level of intellectual disability.

Table 3. Descriptive statistics for Children's Sleep Habits Questionnaire (CSHQ) after multiple imputation, per syndrome.

Syndrome *(N)*	Median *(IQR)*							
	Bedtime Resistance	Sleep Onset Delay	Sleep Duration	Sleep Anxiety	Night Wakings	Parasomnias	SDB	Daytime Sleepiness
**AS *(14)***	6.00 *(4.00)*	1.00 *(2.00)*	5.00 *(3.00)*	5.00 *(3.00)*	6.00 *(2.00)*	10.00 *(2.00)*	3.00 *(1.00)*	10.00 *(4.00)*
CdC *(8)*	9.00 *(6.00)*	2.00 *(1.00)*	3.00 *(1.00)*	6.00 *(1.00)*	5.00 *(2.00)*	9.00 *(3.00)*	3.00 *(0.00)*	9.00 *(1.00)*
**CdLS *(23)***	8.00 *(5.00)*	2.00 *(1.00)*	3.00 *(3.00)*	5.00 *(2.00)*	4.00 *(2.00)*	9.00 *(3.00)*	3.00 *(1.00)*	11.00 *(4.00)*
**CSS *(14)***	7.00 *(4.00)*	1.00 *(0.00)*	3.00 *(1.00)*	6.00 *(2.00)*	3.00 *(3.00)*	10.00 *(4.00)*	4.00 *(2.00)*	11.00 *(2.00)*
DS *(3)*	8.00 *(0.00)*	2.00 *(2.00)*	6.00 *(5.00)*	4.00 *(1.00)*	5.00 *(2.00)*	11.00 *(5.00)*	5.00 *(5.00)*	12.00 *(2.00)*
**FXS *(23)***	7.00 *(4.00)*	1.00 *(1.00)*	3.00 *(3.00)*	6.00 *(5.00)*	4.00 *(2.00)*	9.00 *(4.00)*	3.00 *(2.00)*	11.00 *(2.25)*
JdVS *(9)*	8.00 *(6.00)*	1.00 *(1.00)*	4.00 *(1.00)*	7.00 *(2.00)*	4.00 *(3.00)*	11.00 *(2.00)*	4.00 *(2.25)*	11.00 *(4.00)*
**KBGS *(53)***	9.00 *(5.00)*	1.00 *(1.00)*	4.00 *(3.00)*	5.00 *(3.00)*	5.00 *(3.00)*	10.00 *(4.00)*	3.00 *(1.00)*	12.00 *(3.00)*
**KS *(32)***	7.00 *(4.00)*	1.00 *(1.00)*	3.00 *(3.00)*	6.00 *(3.00)*	5.00 *(3.00)*	10.00 *(2.00)*	3.00 *(1.00)*	10.50 *(4.00)*
PKS *(6)*	6.50 *(1.00)*	2.50 *(1.00)*	5.00 *(3.00)*	4.00 *(1.00)*	5.00 *(1.00)*	11.00 *(4.00)*	4.50 *(2.00)*	15.00 *(3.00)*
**PWS *(16)***	7.00 *(3.00)*	1.00 *(1.00)*	4.00 *(2.00)*	4.00 *(2.00)*	5.00 *(3.00)*	9.00 *(3.00)*	4.00 *(2.00)*	14.00 *(5.00)*
**RTS *(33)***	7.00 *(3.00)*	1.00 *(1.00)*	3.00 *(2.00)*	5.00 *(2.00)*	4.00 *(4.00)*	10.00 *(3.00)*	4.00 *(2.00)*	11.00 *(4.00)*
**SAS *(36)***	8.00 *(4.00)*	1.00 *(1.00)*	4.00 *(3.00)*	6.00 *(4.00)*	5.00 *(3.00)*	11.00 *(3.00)*	3.00 *(1.00)*	12.00 *(3.00)*
**SMS *(17)***	9.00 *(5.00)*	1.00 *(0.00)*	6.00 *(3.00)*	6.00 *(3.00)*	7.00 *(3.00)*	10.00 *(3.00)*	4.00 *(2.00)*	12.00 *(2.00)*
SYNGAP *(4)*	10.00 *(7.00)*	2.00 *(2.00)*	4.00 *(2.00)*	6.00 *(4.00)*	7.00 *(3.00)*	10.00 *(0.25)*	3.00 *(3.00)*	13.00 *(5.00)*
**TSC *(11)***	7.00 *(2.00)*	2.00 *(1.00)*	3.00 *(2.00)*	5.00 *(2.00)*	5.00 *(2.00)*	9.00 *(2.00)*	3.00 *(2.00)*	14.00 *(4.00)*
WHS *(6)*	9.50 *(6.00)*	1.00 *(0.00)*	5.00 *(4.00)*	6.00 *(5.00)*	5.50 *(4.00)*	9.00 *(2.00)*	3.00 *(1.00)*	11.00 *(6.00)*
**WSS *(10)***	10.00 *(7.00)*	2.00 *(1.00)*	5.00 *(4.00)*	7.00 *(2.00)*	4.50 *(5.00)*	10.00 *(3.00)*	4.00 *(2.00)*	11.00 *(3.00)*

Note. AS: Angelman syndrome. CdC: Cri-du-Chat syndrome. CdLS: Cornelia de Lange syndrome. CSS: Coffin-Siris syndrome. DS: Down syndrome. FXS: Fragile X syndrome. IQR: interquartile range. JdVS: Jansen-de Vries syndrome. KBGS: KBG syndrome. KS: Kleefstra syndrome. PKS: Pallister-Killian syndrome. PWS: Prader-Willi syndrome. RTS: Rubinstein-Taybi syndrome. SAS: SATB2-associated syndrome. SMS: Smith-Magenis syndrome. TSC: tuberous sclerosis complex. WHS: Wolf-Hirschhorn syndrome. WSS: Wiedemann-Steiner syndrome.
Syndromes highlighted in bold have N≥10.

Table 4. Odds ratios for sleep difficulties according to the Children's Sleep Habits Questionnaire (CSHQ), after multiple imputation.

	Odds ratio *(95% CI)*							
Syndrome *(N)*	Bedtime Resistance	Sleep Anxiety	Sleep Duration	Night Wakings	Parasomnias	SDB	Daytime Sleepiness	Sleep Onset Delay
AS *(14)*	4.28 *(0.80-22.91)*	1.52 *(0.33-6.90)*	**7.12** ***(2.08-24.34)***	**22.68** ***(5.45-94.38)***	**7.89** ***(1.74-35.84)***	**6.05** ***(1.05-35.00)***	0.48 *(0.08-2.87)*	**6.73** ***(1.61-28.25)***
CdLS *(23)*	**11.54** ***(3.16-42.10)***	0.95 *(0.23-3.90)*	**3.37** ***(1.11-10.26)***	**5.06** ***(1.24-20.67)***	**9.01** ***(2.41-33.62)***	**7.12** ***(1.57-32.19)***	0.51 *(0.13-2.06)*	**4.77** ***(1.30-17.54)***
CSS *(14)*	2.80 *(0.42-18.81)*	1.11 *(0.20-6.15)*	1.56 *(0.31-7.82)*	**8.89** ***(1.94-40.70)***	**17.31** ***(4.04-74.06)***	**19.04** ***(3.96-91.53)***	0.51 *(0.08-3.10)*	1.82 *(0.28-11.78)*
FXS *(23)*	**5.87** ***(1.09-23.01)***	5.22 *(0.54-14.52)*	**3.15** ***(2.35-9.56)***	**6.50** ***(5.88-25.04)***	**9.28** ***(2.14-34.16)***	**10.32** ***(1.43-43.60)***	0.28 *(0.09-1.55)*	0.84 *(0.91-6.21)*
KBGS *(53)*	**13.20** ***(4.21-41.40)***	2.22 *(0.89-5.57)*	**5.11** ***(2.11-12.37)***	**10.58** ***(3.35-33.44)***	**12.90** ***(4.11-40.47)***	**4.97** ***(1.26-19.58)***	0.52 *(0.19-1.46)*	**3.87** ***(1.25-11.96)***
KS *(32)*	**4.76** ***(1.24-18.18)***	2.00 *(0.68-5.89)*	**2.95** ***(1.03-8.47)***	**19.25** ***(5.69-65.18)***	**16.00** ***(4.71-54.35)***	**6.64** ***(1.56-28.29)***	0.46 *(0.12-1.70)*	2.50 *(0.64-9.78)*
PWS *(16)*	3.60 *(0.65-19.84)*	0.68 *(0.10-4.60)*	2.49 *(0.64-9.73)*	**7.23** ***(1.61-32.50)***	**9.43** ***(2.20-40.51)***	**14.13** ***(3.00-66.60)***	1.11 *(0.29-4.24)*	2.34 *(0.41-13.28)*
RTS *(33)*	**5.85** ***(1.62-21.11)***	1.01 *(0.30-3.45)*	2.51 *(0.88-7.19)*	**11.62** ***(3.44-39.29)***	**11.67** ***(3.45-39.45)***	**15.56** ***(4.06-59.60)***	0.59 *(0.18-1.89)*	2.38 *(0.62-9.12)*
SAS *(36)*	**9.25** ***(2.71-31.57)***	2.54 *(0.93-6.93)*	**5.43** ***(2.07-14.22)***	**13.73** ***(4.11-45.80)***	**29.61** ***(8.89-98.60)***	**5.45** ***(1.28-23.29)***	0.97 *(0.35-2.67)*	3.13 *(0.88-11.12)*
SMS *(17)*	**8.13** ***(1.93-34.17)***	1.97 *(0.52-7.39)*	**12.61** ***(3.93-40.46)***	**47.16** ***(11.41-194.83)***	**16.60** ***(4.23-65.19)***	**11.27** ***(2.42-52.54)***	0.50 *(0.10-2.55)*	1.43 *(0.22-9.08)*
TSC *(11)*	3.28 *(0.45-24.04)*	0.93 *(0.14-6.05)*	2.68 *(0.57-12.67)*	**7.43** ***(1.39-39.66)***	**6.64** ***(1.21-36.54)***	**13.25** ***(2.39-73.34)***	1.11 *(0.23-5.35)*	2.13 *(0.27-16.74)*
WSS *(10)*	**13.15** ***(2.72-63.46)***	**7.61** ***(1.94-29.44)***	**6.15** ***(1.54-24.54)***	**13.32** ***(2.76-64.19)***	**9.55** ***(1.89-48.26)***	**11.83** ***(2.09-66.83)***	0.40 *(0.05-3.09)*	**13.67** ***(3.08-60.71)***

Note. AS: Angelman syndrome. CdLS: Cornelia de Lange syndrome. CSS: Coffin-Siris syndrome. FXS: Fragile X syndrome. KBGS: KBG syndrome. KS: Kleefstra syndrome. PWS: Prader-Willi syndrome. RTS: Rubinstein-Taybi syndrome. SAS: SATB2-associated syndrome. SMS: Smith-Magenis syndrome. TSC: tuberous sclerosis complex. WSS: Wiedemann-Steiner syndrome.

Odds ratios are highlighted in bold where 95% CI's did not cross 1.00 threshold.

Table 5. Results from measurement component of the structural equation model, after multiple imputation.

Latent variable	Indicator variable	SMC	Unstd loading	Std loading	SE	*p*
Mental health	ClASP-ID anxiety	0.78	1.00	0.94	-	-
	MIPQ total	0.41	-0.72	-0.52	0.13	<0.001
ADHD characteristics	TAQ impulsivity	0.78	1.00	0.82	-	-
	TAQ overactivity	0.66	0.99	0.79	0.16	<0.001

Note. CIASP-ID: Clinical Anxiety Screen for people with Severe to Profound Intellectual Disability. MIPQ: Mood Interest and Pleasure Questionnaire. SE: standard error. SMC: squared multiple correlation. Std: standardised. TAQ: The Activity Questionnaire. Unstd: unstandardised.
SE and *p* corresponds to the unstandardised loadings.

Table 6. Fitness indices for the structural equation model, after multiple imputation.

Fit indices	Pre-defined threshold	Observed statistic[a]
Comparative fit index	≥0.90	0.91
Root mean square error of approximation	≤0.08	0.06
Standardised root mean square error of approximation	<0.08	0.08
Tucker-Lewis index	≥0.90	0.89
Chi-square	*p*>0.05	*p*<0.001

Note. [a] If the observed statistic falls within the range specified by the threshold, then good fit is evidenced.

Table 7. Regression coefficients from the structural equation model, after multiple imputation.

Dependent variable	Predictor variables	*B*	β	SE	*p*	SMC
BtC	Sleep difficulties	-0.11	-0.08	0.23	0.64	0.39
	Verbal ability	0.06	0.05	0.10	0.56	
	Autism characteristics	-0.05	-0.07	0.17	0.78	
	**Mental health**	**0.81**	**0.77**	**0.44**	**0.04**	
	Health conditions	0.03	0.03	0.09	0.78	
	SES	0.03	0.05	0.13	0.80	
	Age	-0.09	-0.11	0.12	0.43	
	Ethnicity	-0.32	-0.09	0.28	0.25	
	Sex	-0.02	-0.01	0.20	0.93	
Mental health	**Sleep difficulties**	**0.35**	**0.45**	**0.07**	**<0.001**	0.56
	**Autism characteristics**	**0.26**	**0.30**	**0.07**	**<0.001**	
	SES	0.05	0.06	0.07	0.47	
	**Age**	**0.14**	**0.19**	**0.06**	**0.03**	
	Ethnicity	-0.02	-0.01	0.17	0.89	
	Sex	0.04	0.02	0.12	0.77	
ADHD characteristics	**Sleep difficulties**	**0.26**	**0.31**	**0.06**	**<0.001**	0.39
	**Autism characteristics**	**0.22**	**0.31**	**0.06**	**<0.001**	
	SES	0.02	0.03	0.06	0.81	
	**Age**	**0.12**	**0.09**	**0.05**	**0.02**	
	Ethnicity	-0.09	0.02	0.15	0.56	
	**Sex**	**-0.26**	**-0.17**	**0.10**	**0.01**	
Autism characteristics	**Sleep difficulties**	**0.17**	**0.19**	**0.07**	**0.01**	0.13
	SES	-0.10	-0.06	0.08	0.26	
	**Age**	**0.21**	**0.22**	**0.06**	**0.001**	
	Ethnicity	-0.03	0.01	0.19	0.86	
	Sex	-0.06	-0.02	0.13	0.63	
Verbal ability	Sleep difficulties	0.12	0.15	0.09	0.18	0.27
	**Epilepsy**	**-0.36**	**-0.37**	**0.12**	**0.003**	
	SES	-0.01	0.02	0.12	0.91	
	**Age**	**0.31**	**0.29**	**0.09**	**0.001**	
	Ethnicity	-0.14	-0.04	0.26	0.60	
	Sex	0.02	-0.02	0.19	0.94	
Epilepsy	**Sleep difficulties**	**0.18**	**0.18**	**0.10**	**0.05**	0.09
	SES	0.05	0.07	0.14	0.72	
	Age	0.09	0.09	0.11	0.43	
	Ethnicity	0.04	0.04	0.31	0.91	
	Sex	0.36	0.20	0.22	0.10	
Health conditions	**Sleep difficulties**	**0.29**	**0.28**	**0.06**	**<0.001**	0.13
	**SES**	**-0.17**	**-0.19**	**0.08**	**0.04**	
	Age	-0.02	-0.02	0.07	0.74	
	Ethnicity	0.12	0.05	0.18	0.51	
	Sex	0.10	0.08	0.14	0.47	
Sleep difficulties	**SES**	**-0.18**	**-0.20**	**0.08**	**0.02**	0.06
	Age	-0.01	-0.04	0.07	0.87	
	Ethnicity	-0.11	-0.06	0.19	0.57	
	Sex	-0.14	-0.07	0.13	0.30	

Note. SE: standard error. SMC: squared multiple correlation. *B*: unstandardised regression coefficients. β: standardised regression coefficients. *SE* and *p* corresponds to unstandardised regression coefficients. Significant results (p<0.05) are highlighted in bold.

Table 8. Results of the logistic regressions examining different topographies of behaviours that challenge, after multiple imputation.

Dependent variable	Predictor variables	*B*	SE	*p*	R^2
Aggression	Sleep difficulties	0.04	0.04	0.25	0.10
	Verbal ability	0.08	0.07	0.24	
	Autism characteristics	-0.03	0.04	0.41	
	**Anxiety**	**0.14**	**0.04**	**0.002**	
	Depressed mood	-0.004	0.04	0.92	
	Health conditions	-0.02	0.03	0.47	
	SES	0.02	0.04	0.68	
	Age	-0.005	0.03	0.89	
	Ethnicity	-0.03	0.08	0.77	
	Sex	-0.02	0.06	0.74	
Self-injury	Sleep difficulties	0.003	0.04	0.93	0.09
	Verbal ability	0.06	0.07	0.43	
	**Autism characteristics**	**0.10**	**0.04**	**0.02**	
	Anxiety	0.05	0.04	0.16	
	Depressed mood	-0.03	0.04	0.41	
	Health conditions	0.003	0.03	0.94	
	SES	0.02	0.04	0.64	
	Age	-0.03	0.03	0.33	
	Ethnicity	-0.14	0.09	0.11	
	Sex	0.05	0.06	0.41	
Destruction of property	Sleep difficulties	0.02	0.03	0.48	0.14
	Verbal ability	0.07	0.07	0.28	
	Autism characteristics	0.01	0.03	0.73	
	**Anxiety**	**0.15**	**0.05**	**0.003**	
	Depressed mood	0.02	0.04	0.64	
	Health conditions	-0.02	0.03	0.58	
	SES	0.04	0.04	0.35	
	Age	0.03	0.03	0.32	
	Ethnicity	-0.08	0.08	0.33	
	Sex	-0.07	0.06	0.25	

Note. B: unstandardised regression coefficients. SE: standard error.
Significant results following Benjamini-Hochberg correction are highlighted in bold.
Anxiety variable corresponds to CIASP-ID anxiety subscale.
Depressed mood variable corresponds to MIPQ total score.
