# Supplementary material for: The interactions between sleep difficulties and clinical features in children with rare genetic syndromes: a structural equation modelling approach
Source: BMC Pediatr. 2026 Jun 5;26:740. doi: 10.1186/s12887-026-07099-5 (PMC13471272; doi:10.1186/s12887-026-07099-5)
Supplement: Supplementary file 7 [file 12887_2026_7099_MOESM7_ESM.pdf]

Additional file 7: Results of the logistic regressions examining different topographies of behaviours that challenge

Dependent variable	Predictor variables	*B*	SE	*p*	R^2
Aggression	Sleep difficulties	0.008	0.006	0.14	0.19
	Verbal ability	0.03	0.07	0.67	
	Autism characteristics	-0.07	0.04	0.06	
	**Anxiety**[a]	**0.01**	**0.002**	**<0.001**	
	Depressed mood[b]	-0.002	0.01	0.79	
	Health conditions	-0.01	0.01	0.16	
	SES	0.02	0.04	0.54	
	Age	-0.0004	0.0006	0.48	
	Ethnicity	0.03	0.08	0.68	
	Sex	-0.06	0.06	0.36	
Self-injury	Sleep difficulties	0.001	0.006	0.88	0.18
	Verbal ability	0.09	0.07	0.22	
	**Autism characteristics**	**0.13**	**0.04**	**<0.001**	
	Anxiety[a]	0.005	0.002	0.05	
	Depressed mood[b]	-0.006	0.006	0.34	
	Health conditions	-0.0006	0.009	0.94	
	SES	0.07	0.04	0.06	
	Age	-0.001	0.001	0.04	
	Ethnicity	-0.15	0.08	0.07	
	Sex	0.10	0.06	0.12	
Destruction of property	Sleep difficulties	0.003	0.006	0.56	0.23
	Verbal ability	0.07	0.07	0.30	
	Autism characteristics	0.01	0.03	0.69	
	**Anxiety**[a]	**0.01**	**0.002**	**<0.001**	
	Depressed mood[b]	0.007	0.006	0.18	
	Health conditions	-0.005	0.008	0.59	
	SES	0.08	0.03	0.02	
	Age	0.0004	0.0006	0.52	
	Ethnicity	-0.07	0.08	0.39	
	Sex	-0.06	0.06	0.28	

Note. B: unstandardised regression coefficients. SE: standard error.

Significant results following Benjamini-Hochberg correction are highlighted in bold.

[a] Anxiety variable corresponds to CIASP-ID anxiety subscale.

[b] Depressed mood variable corresponds to MIPQ total score.
